# Supplementary material for: Self-assembly of a supramolecular hexagram and a supramolecular pentagram
Source: Nat Commun. 2017 May 19;8:15476. doi: 10.1038/ncomms15476 (PMC5454539; doi:10.1038/ncomms15476)
Supplement: Supplementary Information — Supplementary Figures, Supplementary Methods and Supplementary References [file ncomms15476-s1.pdf]

Supplementary Methods

General Procedures. All starting materials were purchased from Aldrich, Alfa Aesar and used without further purification. Compound **S1-S5, *V*** and ***X*** **(see supplementary Figure 1&2)**were synthesized according to the reported methods[1,2,3], complex (Fe_3***V***$_3$)$^{6+}$ is consistent with the results published by Ludlow III[4]. Column chromatography was conducted by using basic Al_2O_3 (sinopharm chemical reagents co., Ltd, 200-300 mesh) or SiO_2 (Qingdao Haiyang Chemical co., Ltd, 200-300 mesh). The ^{1}H NMR and ^{13}C NMR spectra were recorded on a Bruker Avance 400-MHz, 500-MHz and 600-MHz NMR spectrometer in $CDCl_3$, DMSO-D_6 and CD_3CN with TMS as the inner standard. UV–*vis* absorption spectra were recorded with an Agilent 8453 UV-*vis* Spectrometer. Photoluminescence (PL) spectra were recorded on a Hitachi 2500 Luminescence spectrometer. Transmission Electron Microscopy (TEM) was obtained on JEOL 2010. Electro-spray ionization (ESI) mass spectra were recorded with a Waters Synapt G2 tandem mass spectrometer, using solutions of 0.01mg sample in 1 mL of $CHCl_3$/CH_3OH (1:3, v/v) for ligand or 0.5 mg in 1 mL of MeCN, MeOH or MeCN/MeOH (3:1, v/v) for complex.

TWIM MS. The TWIM MS experiments were performed under the following conditions: ESI capillary voltage, 3 kV; sample cone voltage, 30 V; extraction cone voltage, 3.5 V; source temperature 100 ℃; desolvation temperature, 100 ℃; cone gas flow, 10 L/h; desolvation gas flow, 700 L/h (N_2); source gas control, 0 mL/min; trap gas control, 2 mL/min; Helium cell gas control, 100 mL/min; ion mobility (IM) cell gas control, 30 mL/min; sample flow rate, 5 µL/min; IM traveling wave height, 25 V; and IM traveling wave velocity, 1000 m/s. Q was set in rf-only mode to transmit all ions produced by ESI into the tri-wave region for the acquisition of TWIM MS data.

Molecular Modeling: Energy minimization of the macrocycles was conducted with the Materials Studio version 6.0 program, using Anneal and Geometry Optimization tasks in the Materials Studio Forcite module (Accelrys, Inc.).

STM: The sample was dissolved in DMF or CH_3CN at a concentration of 5.0 mg/ml. Solution (5ul) was dropped on HOPG surface. After 30 seconds, surface was washed slightly with water for three times and totally dried in R.T. in air. The STM images were taken with a PicoPlus SPM system with a PicoScan 3000 Controller. The obtained STM images were processed by WSxM software.[S6]

AFM: The 40N/m rectangle AFM tip was used to make markers on the surface of the sample with nanoshaving technique. The size of the markers is 10µm×10µm and the distances between two closest markers are 30µm. The loading forces used are 6µN and shaving speed is around 5µm/s.

Synthesis of the ligands and complexes

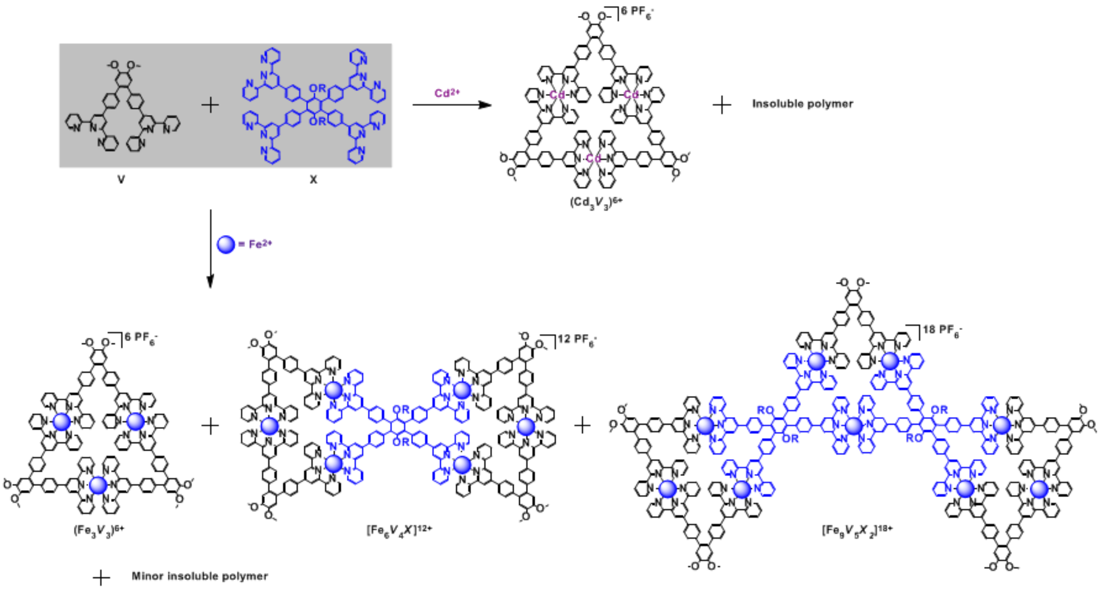

Supplementary Figure 1: Directly self-assembly of organic ligands *V* and *X* with Cd^{2+} or Fe^{2+}, respectively. *V*+*X*+Cd^{2+} only produced a metallo-triangle and the unidentified insoluble polymer; however, *V*+*X*+Fe^{2+} generated two separated major complexes, metallo-triangle and metallo-bowtie, and trace amount of tri-metallo-triangle, as well small amount of insoluble polymer.

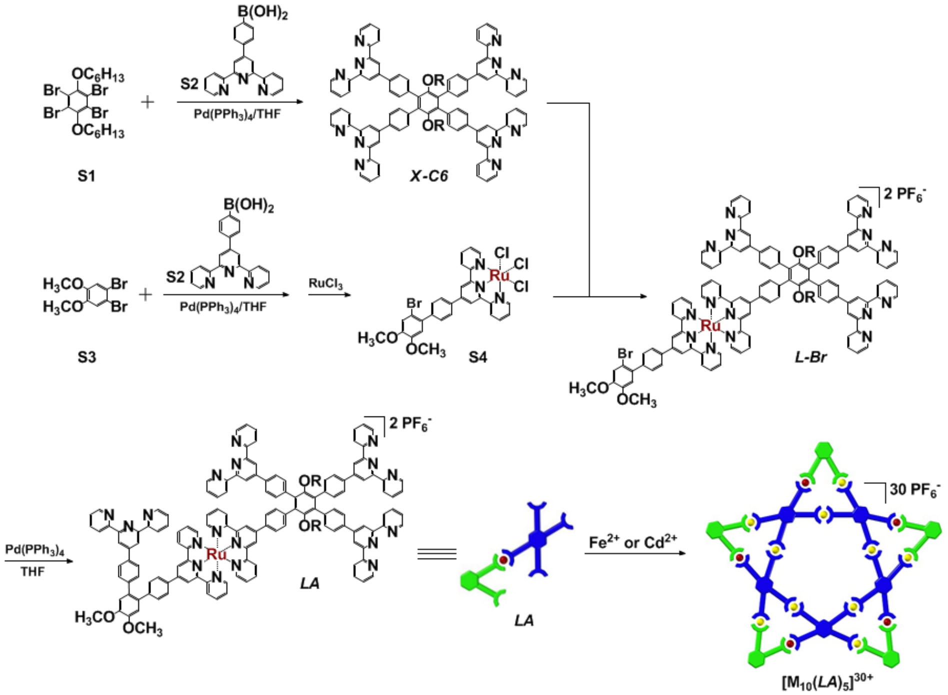

Supplementary Figure 2: Synthetic route of supramolecular dinuclear metallo-pentagram *via* predesigned terpyridinyl metallo-organic ligand ***LA***.

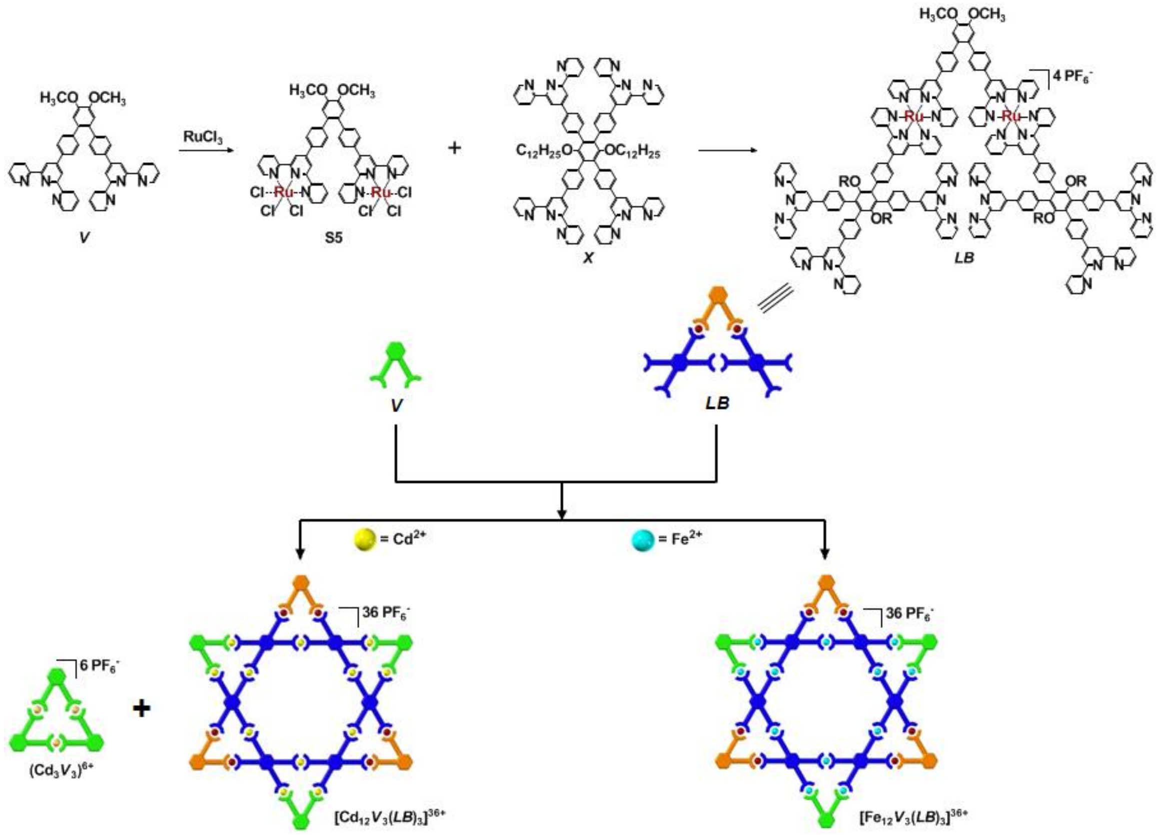

Supplementary Figure 3: Synthetic route of supramolecular six-star shaped hexagram *via* terpyridinyl metallo-organic ligand ***LB***.

Preparation of Ligands and Supramolecular architectures.

1. Compound monobromoterpyridine:

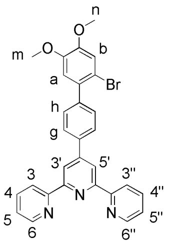

To a solution of 1, 2-dibromo-4,5-dimethoxybenzene (1.48 g, 5.0 mmol) and 4'-(4-boronatophenyl) [2,2':6',2"]terpyridine (S3, 1.78 g, 5.00 mmol) in THF (200 mL), aq. Na_2CO_3 (20 mL, 1 M) was added. The mixture was freeze-pump-thawed three times and backfilled with argon; then $Pd(PPh_3)_4$ (100 mg) was added. After refluxing for 12 hours under argon, the mixture was cooled to 25 ℃ and poured into aq. NH_4Cl solution. The aqueous layer was extracted with $CHCl_3$, and then the combined organic phase was washed with brine and dried over $MgSO_4$. After concentration in vacuum, the residue was purified by flash column chromatography (Al_2O_3, 200-300 mesh), eluting with $CHCl_3$:Petrol (2:1) to get monobromo-terpyridine as a white solid: 0.66g (25%); m.p.= 218 ℃; ^{1}H NMR (400 MHz, $CDCl_3$) δ 8.80 (s, 2H, 3′,5′-tpy*H*), 8.75 (m, 2H, 6,6″-tpy*H*), 8.69 (m, 2H, 3,3″-tpy*H*), 7.99 (d, J = 8.3 Hz, 2H, Ar*H* ^{g}), 7.88 (td, J = 7.8, 1.7 Hz, 2H, 5,5″-tpy*H*), 7.70 (d, J = 8.3 Hz, 2H, Ar*H* ^{g}), 7.39 (m, 4.8, 2H, 4,4″-tpy*H*), 6.98 (s, 1H, ArH^b), 6.96 (s, 1H, ArH^a), 4.00 (s, 3H, -OMe^m), 3.94 (s, 3H, -OMe^n). ^{13}C NMR (100 MHz, $CDCl_3$) δ 156.27, 155.95, 149.73, 149.11, 141.65, 136.83, 127.25, 123.84, 121.39, 119.51, 118.61, 111.54, 110.32, 77.41, 77.10, 76.78, 56.01. ESI/MS (solvent: $CHCl_3$: MeOH=1:3, m/z): 524.2 $[M+H]^+$, Calculated $[M+H]^+$: 524.1.

2. Complex S4

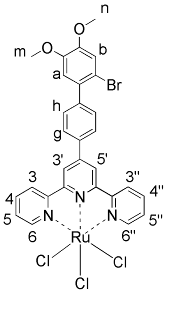

Compound monobromo-terpyridine (262 mg, 0.5 mmol) and $RuCl_3 \cdot 3H_2O$ (148 mg, 0.55 mmol) was suspended in EtOH (100 mL), The mixture were refluxed for 12 hours, then cooled to 25 ℃ and filtered to obtain a brown powder. The solid was washed with MeOH repeatedly until the filtrate get clean and colorless, the solid was collected and dried in vacuum for 12 hours to get the 200 mg desired red powder with the yield of 75%, it was used directly for the next step without further purification. m.p.>320 ℃; Elemental Anal. Calcd. for $C_{29}H_{22}BrN_3O_2RuCl_3 \cdot 3H_2O$: C, 44.32; H, 3.59; N, 5.35. Found: C, 43.98; H, 3.62; N, 5.31.

3. Complex *L-Br*

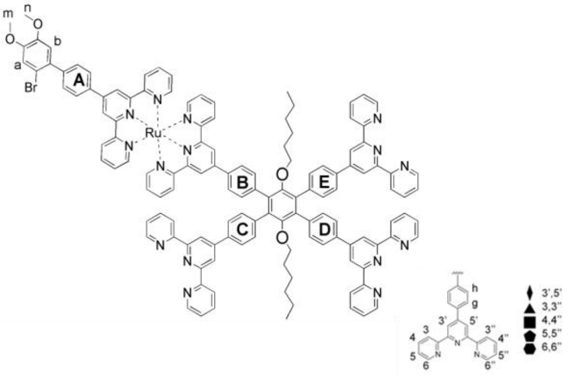

To a solution of **S4** (36.1 mg, 0.05 mmol) and ***X*** (150.6 mg, 0.1 mmol) in $CHCl_3$/MeOH (100 mL, 1:1 v/v) together with 5 drops of N-ethylmorpholine as a reducing agent, the mixture was refluxed for 12 hours. The mixture was cooled to 25 ℃ and the solvent was removed under vacuum. The residue was extracted with $CHCl_3$, and then the combined organic phase was washed with brine and dried over $MgSO_4$, After concentration in vacuum, the residue was purified by flash column chromatography [Al_2O_3 200-300 mesh, eluting with $CHCl_3$:MeOH (40:1, v/v)] to get ***L-Br*** as a red powder: 55mg (yield: 50%); m.p. >320 ℃; ^{1}H NMR (500 MHz, $CDCl_3$) δ 9.49 (s, tpy^A$H^{3',5}$,2H), 9.24 (d, J = 8.3 Hz, tpy^A$H^{3,3''}$, 2H), 9.16 (s, tpy^B$H^{3',5'}$, 2H), 8.91 (d, J = 8.2 Hz, tpy^B$H^{3,3''}$, 2H), 8.78-8.76 (m, 6H, tpy^C$H^{3',5'}$, tpy^D$H^{3',5'}$, tpy^E$H^{3',5}$), 8.70 (m, tpy^C $H^{6,6'',}$ tpy^D $H^{6,6'',}$ tpy^E $H^{6,6''}$, 6H), 8.66 (m, tpy^C$H^{3,3''}$, tpy^D$H^{3,3''}$, tpy^E$H^{3,3''}$, 6H), 8.56 (d, J = 8.3 Hz, tpy^A H^g, 2H), 8.25 (d, J = 8.3 Hz, tpy^BH^g,2H), 7.97 (m, tpy^A$H^{4,4''}$, tpy^B$H^{4,4''}$, 4H), 7.87 (m, tpy^C H^h , tpy^D H^h, tpy^E H^h, tpy^C$H^{4,4''}$, tpy^D$H^{4,4''}$, tpy^E$H^{4,4''}$, 12H), 7.80 (d, J = 8.3 Hz, tpy^A H^h 2H), 7.71 (d, J = 8.2 Hz, tpy^b H^h , 2H), 7.54 (d, J = 8.3 Hz, tpy^C H^g 2H), 7.50 (m, tpy^D H^g , tpy^E H^g 4H), 7.45 (d, J = 5.0 Hz, tpy^A $H^{6,6''}$2H), 7.40 (d, J = 5.1 Hz, tpy^C $H^{6,6''}$, 2H), 7.34 (m, tpy^B $H^{6,6''}$, tpy^C $H^{6,6''}$, tpy^D $H^{6,6''}$, tpy^E $H^{6,6''}$, tpy^F $H^{6,6'}$, '8H), 7.21 (dd, J = 11.8, 4.7 Hz, tpy^A $H^{6,6''}$2H), 7.19 (s, tpy^A H^J, 1H), 7.00 (s, tpy^A H^K, 1H), 3.98 (s, H^{OMe} , 3H), 3.96 (s, H^{OMe} ,3H), 3.18 (t, J = 6.1 Hz, H^{OCH_2R}, 2H), 3.14 (t, J = 6.2 Hz, H^{OCH_2R} , 2H), 1.30 (m, 6H), 1.02 (m, 4H), 0.85 (m, 6H), 0.63 (dt, J = 16.4, 7.3 Hz, 6H). ^{13}C NMR (125 MHz, $CDCl_3$) δ 158.21, 157.95, 156.55, 155.85, 155.10, 155.10, 152.08, 151.35, 149.07, 148.97, 138.52, 138.24, 137.75, 137.04, 136.77, 136.49, 136.13, 135.56, 135.06, 132.86, 132.61, 132.18, 131.86, 131.00, 129.66, 128.05, 127.85, 126.95, 126.32, 125.84, 125.30, 123.91, 123.67, 122.42, 121.80, 121.49, 121.31, 118.88, 56.33, 31.40, 31.33, 29.84, 29.69, 29.35, 29.24, 25.40, 25.29, 22.68, 22.58, 22.47, 14.03, 13.96, 13.87. HR-MS (solvent: $CHCl_3$: MeOH=1:3, m/z): 1066.80 $[M-2Cl]^{2+}$, calculated: $[M-2Cl]^{2+}$=1066.65, 711.53$[M+H-2Cl]^{3+}$, calculated: $[M+H-2Cl]^{3+}$=711.43.

4. Complex *LA*:

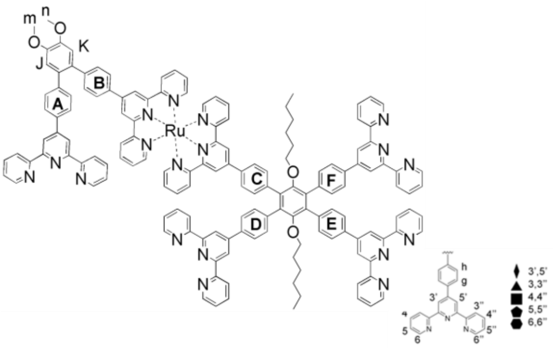

To a solution of ***L-Br*** (21.3 mg, 0.01 mmol) and 4'-(4-boronatophenyl) [2, 2':6', 2"] terpyridine (**S2,** 35.4 mg, 0.1 mmol) in DMSO (20 mL), aq. Na_2CO_3 (0.5 mL, 1 M) was added. The mixture was freeze-pump-thawed three times and backfilled with argon; then $Pd(PPh_3)_4$ (5 mg) was added. After refluxing for 24 hours under argon, the mixture was cooled to 25 ℃ and poured into an aq. NH_4Cl solution. The aqueous layer was extracted with $CHCl_3$, and then the combined organic phase was washed with brine and dried over $MgSO_4$. After concentration in vacuum, the residue was purified by flash column chromatography [Al_2O_3, 200-300 mesh, eluting with $CHCl_3$:MeOH (40:1)] to get ***LA*** as a red solid: 12 mg (yield: 48%). m. p. >320 ℃; ^{1}H NMR (500 MHz, $CDCl_3$) δ 9.28 (s, $tpy^{A}H^{3',5'}$, 2H), 9.08 (s, $tpy^{B}H^{3',5'}$, 2H), 9.04 (d, J = 8.4 Hz, $tpy^{A}H^{3,3''}$, 2H), 8.83 (d, J = 8.0 Hz, $tpy^{B}H^{3,3''}$ 2H), 8.75 (m, $tpy^{C}H^{3',5'}$, $tpy^{D}H^{3',5'}$, $tpy^{E}H^{3',5'}$, $tpy^{F}H^{3',5'}$, 8H), 8.70 (m, tpy^{C} $H^{6,6''}$, tpy^{D} $H^{6,6''}$,4H), 8.65 (m, tpy^{E} $H^{6,6''}$, tpy^{F} $H^{6,6''}$, $tpy^{C}H^{3,3''}$, $tpy^{D}H^{3,3''}$, $tpy^{E}H^{3,3''}$, $tpy^{F}H^{3,3''}$, 12H), 8.33 (d, J = 8.3 Hz, tpy^{A} H^{g} 2H), 8.23 (d, J = 8.2 Hz, tpy^{B} H^{g} , 2H), 7.88 (m, $tpy^{A}H^{4,4''}$, $tpy^{B}H^{4,4''}$, $tpy^{C}H^{4,4''}$, $tpy^{D}H^{4,4''}$, $tpy^{E}H^{4,4''}$, $tpy^{F}H^{4,4''}$, tpy^{C} H^{g}, tpy^{D} H^{g}, tpy^{E} H^{g}, tpy^{F} H^{g}, 18H), 7.70 (d, J = 8.2 Hz, tpy^{A} H^{h} , 2H), 7.54 (t, J = 8.4 Hz, tpy^{B} H^{h}, 4H), 7.50 (m, tpy^{C} H^{h}, tpy^{D} H^{h}, tpy^{E} H^{h}, 8H), 7.37 (m, tpy^{A} $H^{5,5''}$, tpy^{B} $H^{5,5''}$, tpy^{C} $H^{5,5''}$, tpy^{D} $H^{5,5''}$, tpy^{E} $H^{5,5''}$, tpy^{F} $H^{5,5'}$,12H), 7.18 (m, tpy^{A} $H^{6,6''}$, tpy^{B} $H^{6,6''}$, 4H), 7.15 (s, tpy^{A} H^{m}, 1H), 7.06 (s, tpy^{A} H^{n}, 1H), 6.93 (d, J = 8.4 Hz, tpy^{F} H^{h}, 2H), 4.06 (s, H^{OMe}, 3H), 4.04 (s, H^{OMe} , 3H), 3.18 (t, J = 6.1 Hz, H^{OCH_2R} , 2H), 3.13 (t, J = 6.1 Hz, H^{OCH_2R}, 2H), 1.30 (m, 11H), 1.00 (m, 6H), 0.85 (m, 10H), 0.61 (dd, J = 13.8, 7.2 Hz, 6H). ^{13}C NMR (125 MHz, $CDCl_3$) δ 158.68 , 158.11, 157.94, 156.37, 156.14, 155.95, 155.82, 155.61, 154.95, 151.74, 151.19, 151.06, 150.01, 149.78, 149.06, 148.98, 148.87, 148.82, 148.70, 138.29, 138.10, 137.74, 137.07, 136.93, 136.79, 136.42, 136.06, 135.89, 135.59, 135.11, 133.95, 132.88, 132.53, 132.23, 132.11, 131.98, 131.38, 130.72, 127.84, 127.84, 127.00, 126.42, 125.69, 125.26, 123.90, 123.68, 121.76, 121.48, 121.32, 118.89, 118.68, 117.88, 116.36, 113.72, 56.35, 56.22, 31.43, 31.38, 31.33, 30.20, 29.80, 29.73, 29.68, 25.38, 25.32, 22.67, 22.56, 22.46, 14.09, 14.00, 13.86. HR-MS (solvent: $CHCl_3$:MeOH=1:3, m/z): 1180.95 $[M-2Cl]^{2+}$, calculated:

$[M\text{-}2Cl]^{2+}$=1180.88, 787.63 $[M+H\text{-}2Cl]^{3+}$, calculated: $[M+H\text{-}2Cl]^{3+}$= 787.58.

5. Complex *LB*

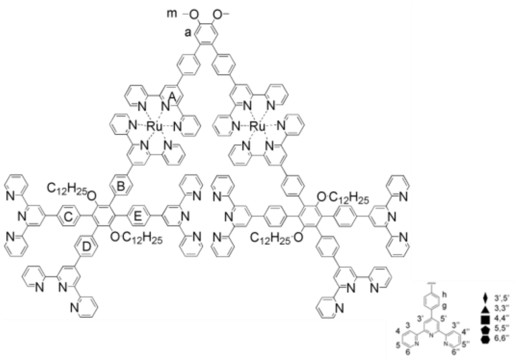

To a suspension of **S5** (29.1 mg, 0.025 mmol) and ***X*** (125.7 mg, 0.075 mmol) in $CHCl_3$/MeOH (250 mL, 1:1 v/v), with 5 drops of N-methylmorpholine as a reducing agent, the mixture was refluxed for 12 hours. The mixture was cooled to 25 ℃ and the solvent was removed under vacuum. The residue was extracted on with $CHCl_3$ for 3 times, and then the combined organic phase was washed with brine and dried over $MgSO_4$. After concentration in vacuum, then purified by flash column chromatography [Al_2O_3, 200-300 mesh, eluting with $CHCl_3$:MeOH (20:1)] to get ***LB*** as a red powder: 32 mg (yield: 30%); m.p. >320 ℃; ^{1}H NMR (500 MHz, $CDCl_3$) δ 9.52 (s, $tpy^{A}H^{3',5'}$, 4H), 9.35 (d, $tpy^{A}H^{3,3''}$, J = 8.2 Hz, 4H), 9.08 (s, $tpy^{B}H^{3',5'}$, 4H), 8.83 (d, $tpy^{B}H^{3,3''}$, J = 8.3 Hz, 4H), 8.76 (m, $tpy^{C}H^{3',5'}$, $tpy^{D}H^{3',5'}$, $tpy^{E}H^{3',5'}$, 12H), 8.70 (m, J = 4.0 Hz, $tpy^{C}H^{6,6''}$, $tpy^{D}H^{6,6''}$, $tpy^{E}H^{6,6'',}$ 12H), 8.65 (m, $tpy^{C}H^{3,3''}$, $tpy^{D}H^{3,3''}$, $tpy^{E}H^{3,3''}$ 12H), 8.35 (d, J = 8.2 Hz, $tpy^{A}H^{g}$, 4H), 8.19 (d, J = 8.2 Hz, $tpy^{B}H^{g}$, 4H), 7.95 (m, tpy^{A} $H^{5,5''}$, tpy^{B} $H^{5,5''}$, 8H), 7.86 (m, $tpy^{C}H^{g}$, $tpy^{D}H^{g}$, $tpy^{E}H^{g}$, tpy^{C} $H^{5,5''}$, tpy^{D} $H^{5,5''}$, tpy^{E} $H^{5,5''}$, 24H), 7.70 (d, J = 8.2 Hz, $tpy^{A}H^{h}$, 4H), 7.58 (d, J = 8.2 Hz, $tpy^{B}H^{h}$, 4H)7.52 (m, $tpy^{C}H^{h}$, $tpy^{D}H^{h}$, $tpy^{E}H^{h}$, 12H), 7.49 (m, $tpy^{A}H^{3,3''}$, $tpy^{B}H^{3,3''}$, 8H), 7.42 (m, $tpy^{A}H^{6,6'',}$ 4H), 7.33 (m, $tpy^{B}H^{6,6'',}$ $tpy^{C}H^{5,5''}$, $tpy^{D}H^{5,5''}$, $tpy^{E}H^{5,5''}$, 16H), 7.25 (m, $tpy^{A}H^{5,5''}$, 4H), 7.15 (m, $tpy^{b}H^{5,5''}$, $tpy^{A}H^{m}$, 6H), 4.05 (s, OMe, 6H), 3.18 (s, OCH_2R, 4H), 3.13 (s, OCH_2R, 2H), 1.30 (dt, J = 34.0, 16.9 Hz, 12H), 1.11 (ddd, J = 23.3, 14.3, 7.0 Hz, 3H), 0.93 (m, 21H), 0.76 (dt, J = 18.2, 7.3 Hz, 6H). ^{13}C NMR (125 MHz, $CDCl_3$) δ 158.32, 157.68, 156.35, 156.10, 155.81, 155.17, 155.00, 151.20, 149.92, 149.03, 148.93, 139.27, 137.72, 137.14, 136.77, 136.40, 135.93, 135.62, 135.00, 132.93, 131.98, 131.27, 128.80, 128.31, 126.41, 123.96, 123.67, 122.58, 121.51, 121.31, 118.82, 118.78, 118.64, 114.05, 56.36, 33.80, 31.91, 31.77, 31.75, 31.50, 31.43, 31.37, 30.20, 30.14, 29.86, 29.68, 29.56, 29.53, 29.47, 29.34, 29.24, 29.19, 29.14, 28.95, 27.21, 25.70, 25.66, 23.77, 22.97, 22.67, 22.58, 22.55, 14.08, 14.03, 10.94. HR-MS(solvent: $CHCl_3$:MeOH=1:3, m/z): 1447.60 $[M\text{-}3Cl]^{3+}$, calculated: $[M\text{-}3Cl]^{3+}$: 1447.57, $[M\text{-}3Cl+H]^{4+}$: 1085.97, calculated: $[M\text{-}3Cl+H]^{4+}$: 1085.93; $[M\text{-}4Cl+H]^{5+}$: 861.78, calculated: $[M\text{-}4Cl+H]^{5+}$: 861.66; $[M\text{-}4Cl+2H]^{6+}$: 718.14, calculated: $[M\text{-}4Cl+2H]^{6+}$: 717.88.

6. Complex of ligand ***V*** and ***X*** with **Fe^{2+}:**

Ligand ***V*** (15.2 mg, 17.0 μmol), ***X*** (25.6 mg, 17.0 μmol) and $FeSO_4 \cdot 7H_2O$ (14.1 mg, 51.0 μmol) were dissolved in 40 Ml ethylene glycol. The solution was heated at 140°C for 2 days under N_2 protection. The solution was cooled down and added into a 60 Ml methanol solution with 1.0 g NH_4PF_6. The precipitate was filtered and residue was flash column chromatographed (SiO_2) eluting with MeCN/sat. KNO_3 (aq) (100:0-100:12) to generate three major fractions as the purple precipitates, after then the counterion was exchanged to PF_6^- again.

6.1. Metallo-triangle $(Fe_3\boldsymbol{V}_3)^{6+}$:[4]

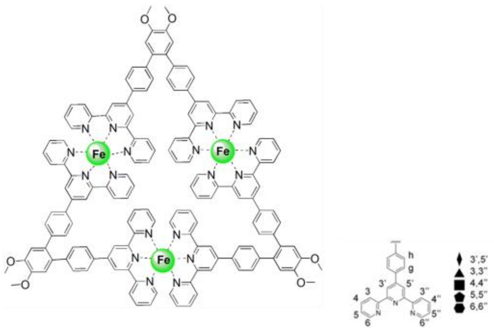

m. p. > 320°C, ^1H NMR (400 MHz, CD_3CN) δ 9.19 (s, 4H, tpy-$H^{3',5'}$), 8.61 (d, J = 7.2 Hz, 4H, tpy-$H^{3,3''}$), 8.27 (d, J = 7.5 Hz, 4H, Ph-H^{g}), 7.82 (d, J = 6.9 Hz, 4H, tpy-$H^{4,4''}$), 7.71 (d, J = 6.8 Hz, 4H, Ph-H^{h}), 7.32 (s, 2H, Ph-H^{m}), 7.22 (d, J = 31.9 Hz, 4H, tpy-$H^{6,6''}$), 7.04 (m, 4H, tpy-$H^{5,5''}$), 4.03 (d, J = 23.3 Hz, 6H, -OCH_3). ^{13}C NMR (100 MHz, CD_3CN) δ 160.25, 158.04, 153.01, 149.86, 149.35, 143.90, 138.66, 134.81, 132.08, 131.45, 127.57, 127.33, 123.86, 121.46, 114.22, 63.17. ESI MS (CD_3CN as solvent , m/z): 1502.8 $[M-2PF_6]^{2+}$ (calcd m/z: 1502.8), 953.7 $[M-3PF_6]^{3+}$ (calcd m/z: 953.7), 678.9 $[M-4PF_6]^{4+}$ (calcd m/z: 678.9), 514.2 $[M-5PF_6]^{5+}$ (calcd m/z: 514.2) and 404.4 $[M-6PF_6]^{6+}$ (calcd m/z: 404.4).

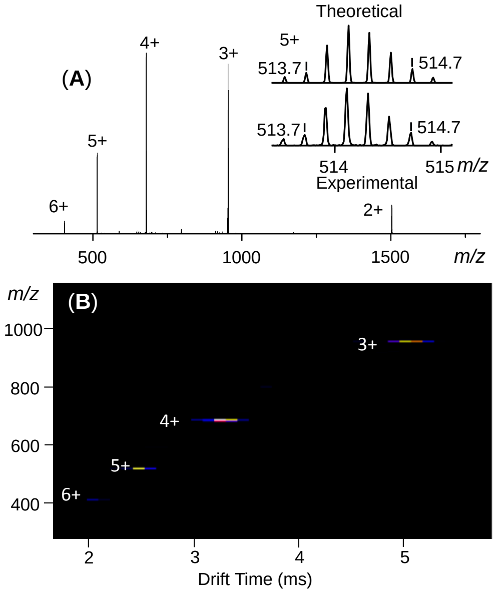

Supplementary Figure 3.1: (A) ESI-MS and (B) 2D TWIM-MS plot (m/z vs drift time) of

[**Fe$_3$*V*$_3$**]$^{6+}$ (PF_6^- as counterion). The charge states of intact assemblies are marked.

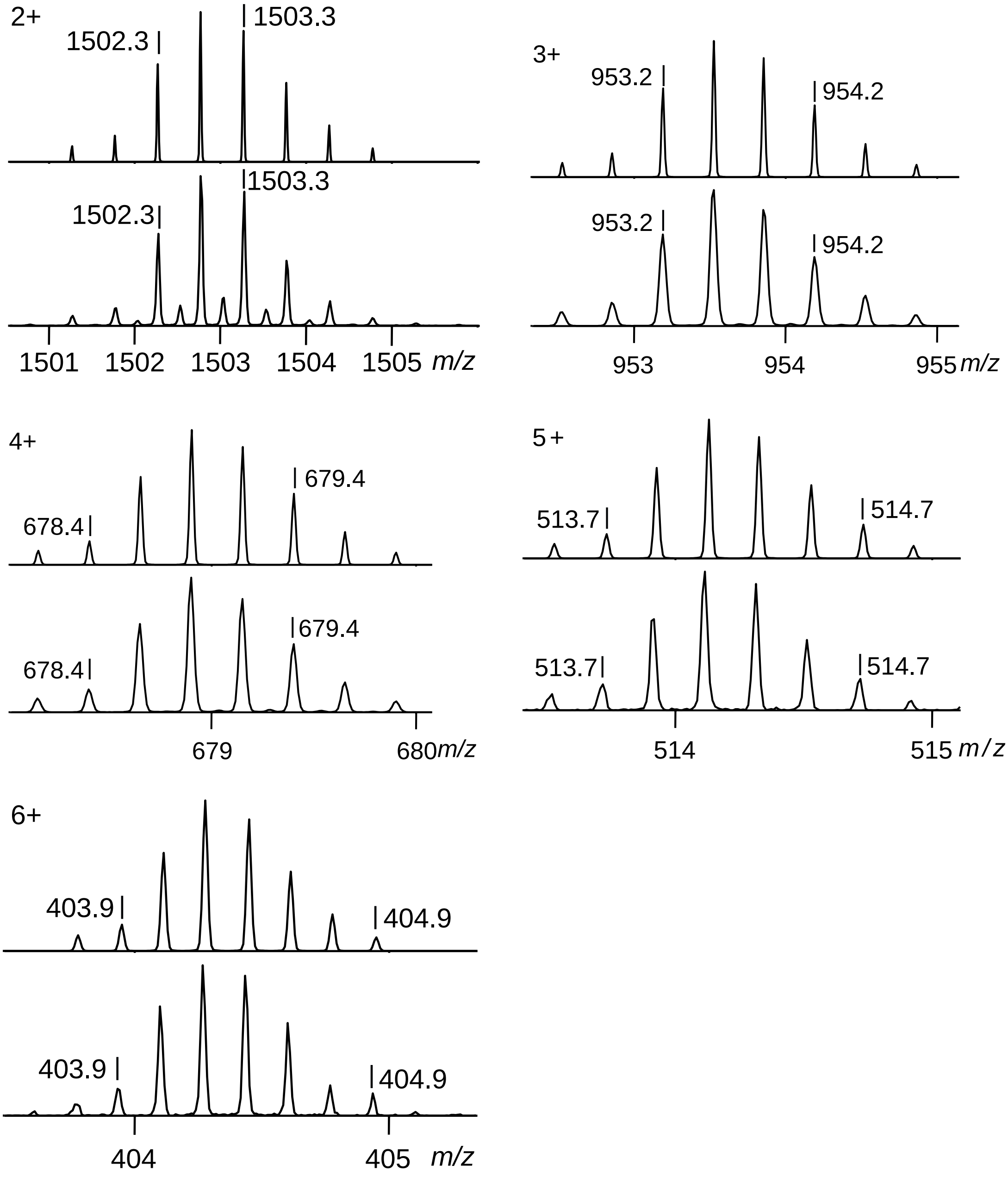

Supplementary Figure 4: Measured (bottom) and calculated (top) isotope patterns for different charge states observed from [**Fe$_3$*V*$_3$**]$^{6+}$ (PF_6^- as counterion, CD_3CN as solvent).

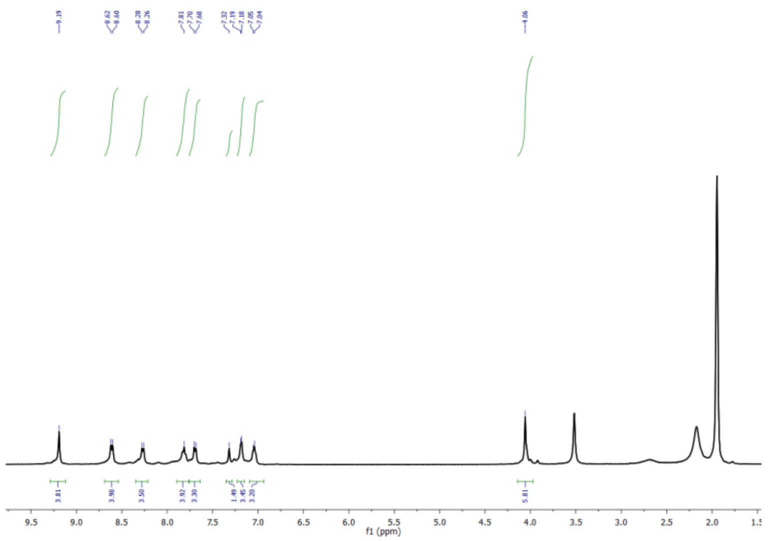

Supplementary Figure 5. ^{1}H NMR (400 MHz, CD_3CN as solvent) spectrum of complex $[Fe_3\boldsymbol{V}_3]^{6+}$ (PF_6^- as counterion).

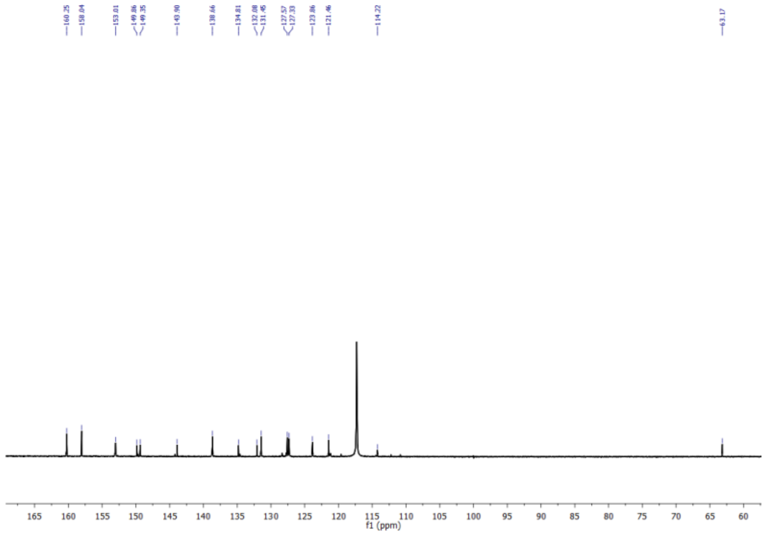

Supplementary Figure 6. ^{13}C NMR (400 MHz, CD_3CN as solvent) spectrum of complex $[\mathbf{Fe_3}\boldsymbol{V}_3]^{6+}$ (PF_6^- as counterion).

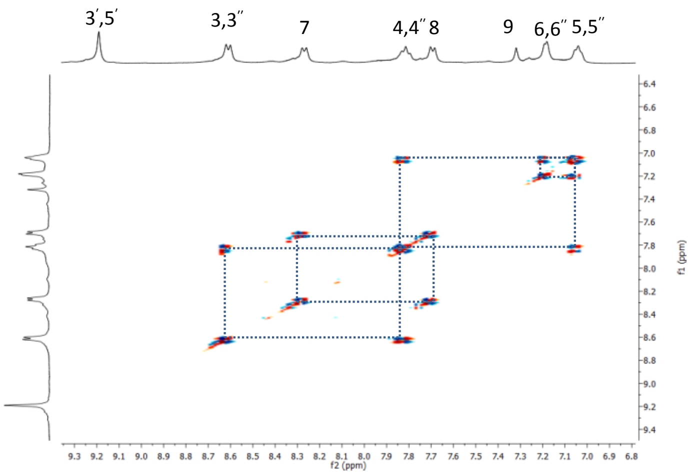

Supplementary Figure 7. 2D COSY NMR (400 MHz, CD_3CN) spectrum of complex $[\mathbf{Fe_3}\boldsymbol{V_3}]^{6+}$ (PF_6^- as counterion) (aromatic region).

2.6.2. Metallo-bowtie $[Fe_6\boldsymbol{V_4X}]^{12+}$:[4]

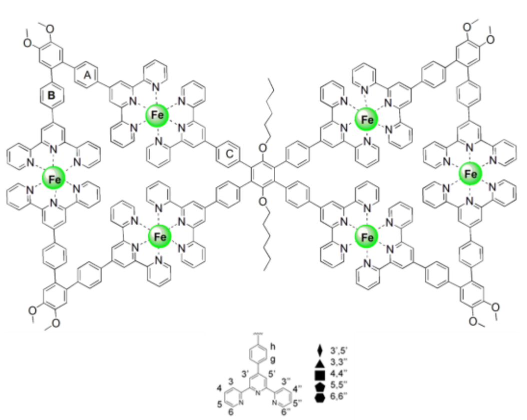

m.p.: > 320°C; ^{1}H NMR (600 MHz, CD_3CN) δ 9.26 (s, 4H, tpy-$H^{3',5'}$), 9.25 (s, 4H, tpy-$H^{a3',5'}$), 9.24 (s, 4H, tpy-$H^{b3',5'}$), 8.66 (m, 12H, tpy-$H^{3,3''}$, tpy-$H^{a3,3''}$ and tpy-$H^{b3,3''}$), 8.42 (d, J = 7.9 Hz, 4H, Ph-H^{7}), 8.30 (d, J = 8.2 Hz, 8H, Ph-H^{a7} and Ph-H^{b7}), 7.93 (d, J = 8.2 Hz, 4H, Ph-H^{8}), 7.85 (m, 12H, tpy-$H^{4,4''}$, tpy-$H^{a4,4''}$ and tpy-$H^{b4,4''}$), 7.73 (dd, J = 8.1, 2.2 Hz, 8H, Ph-H^{a8} and Ph-H^{b8}), 7.35 (d, J = 5.3 Hz, 4H, Ph-H^{a9} and Ph-H^{b9}), 7.23 (d, J = 5.6 Hz, 8H, tpy-$H^{a6,6''}$ and tpy-$H^{b6,6''}$), 7.21 (d, J = 5.6 Hz, 4H, tpy-$H^{6,6''}$), 7.09 (m, 8H, tpy-$H^{a5,5''}$ and tpy-$H^{b5,5''}$), 7.07 – 7.04 (m, 4H, tpy-$H^{5,5''}$), 4.10 (s, 12H, -OCH_3-H^{10}), 3.49 (t, J = 4.8 Hz, 4H, Alkyl-H^{11}), 1.24 (m, 8H, Alkyl-H^{12} and Alkyl-H^{15}), 1.10 – 1.02 (m, 8H, Alkyl-H^{13} and Alkyl-H^{14}), 0.80 (t, J = 7.3 Hz, 6H, Alkyl-H^{16}). ^{13}C NMR (150 MHz, CD_3CN) δ 160.33, 160.26, 160.21, 158.03, 153.01, 151.36, 149.92, 149.69, 149.34, 143.91, 143.85, 139.43, 138.67, 135.96, 135.12, 134.87, 134.78, 132.76, 132.03, 132.00, 131.47, 127.60, 127.54, 127.32, 127.05, 123.88, 121.54, 121.52, 121.30, 114.17, 73.45, 55.82, 31.25, 29.68, 25.37, 22.56, 13.56. ESI MS (CD_3CN as solvent, m/z): 1503.5 $[M\text{-}4PF_6]^{4+}$ (calcd m/z: 1503.5), 1173.9 $[M\text{-}5PF_6]^{5+}$ (calcd m/z: 1173.9), 954.0 $[M\text{-}6PF_6]^{6+}$ (calcd m/z: 954.0), 797.1 $[M\text{-}7PF_6]^{7+}$ (calcd m/z: 797.1), 679.3 $[M\text{-}8PF_6]^{8+}$ (calcd m/z: 679.3), 587.8 $[M\text{-}9PF_6]^{9+}$ (calcd m/z: 587.8), 514.4 $[M\text{-}10PF_6]^{10+}$ (calcd m/z: 514.4)

and 454.6 [M-11PF$_6^-$]$^{11+}$ (calcd *m/z*: 454.6).

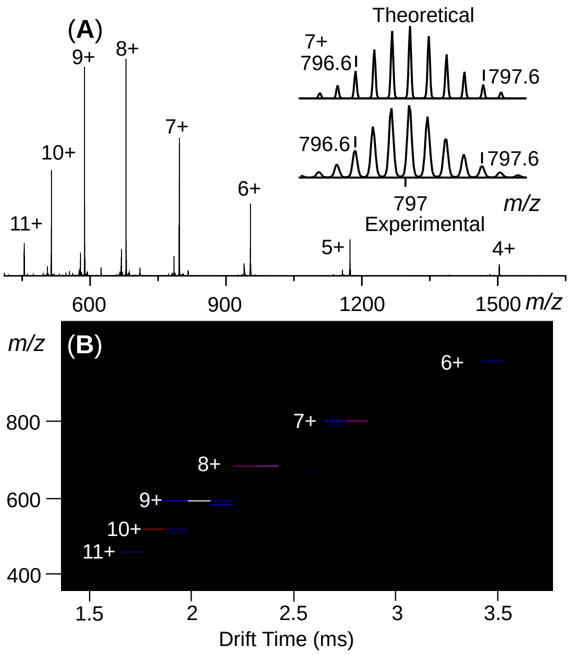

Supplementary Figure 8: (A) ESI-MS and (B) 2D TWIM-MS plot (*m/z* vs drift time) of metallo-bowtie [Fe$_6$***V***$_4$***X***]$^{12+}$. The charge states of intact assemblies are marked.

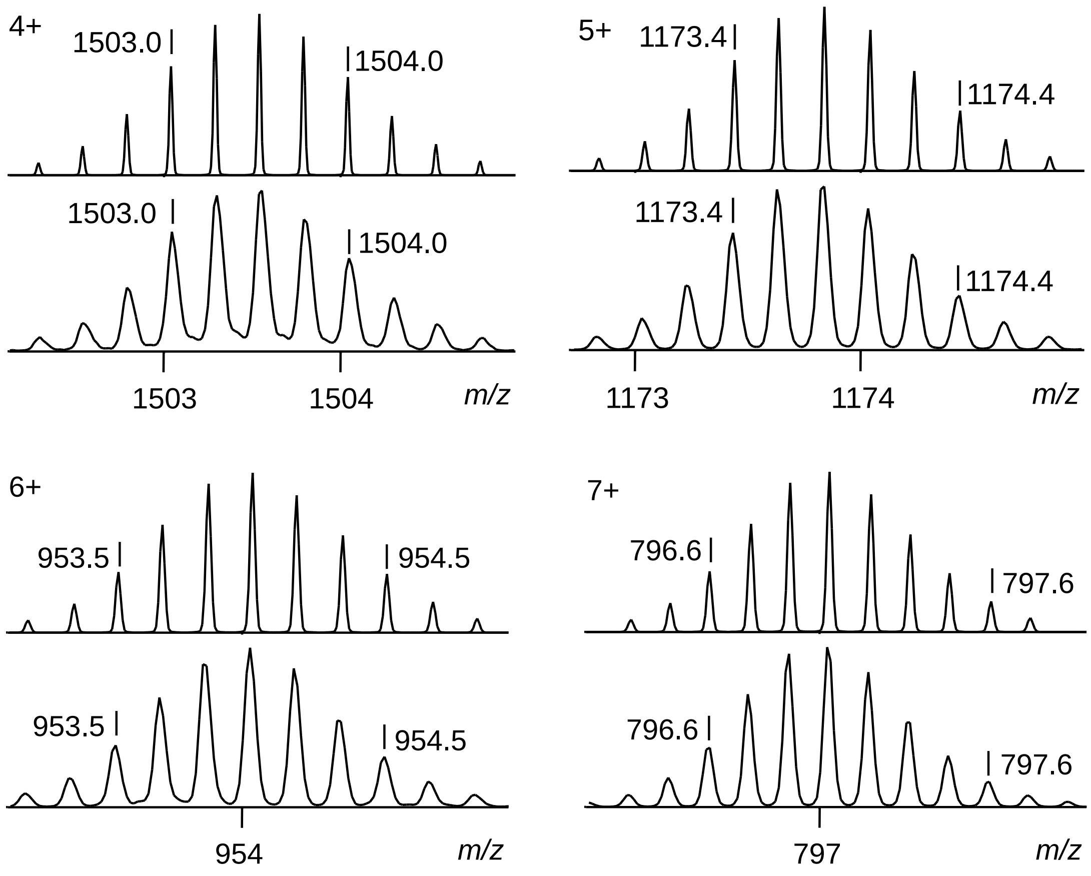

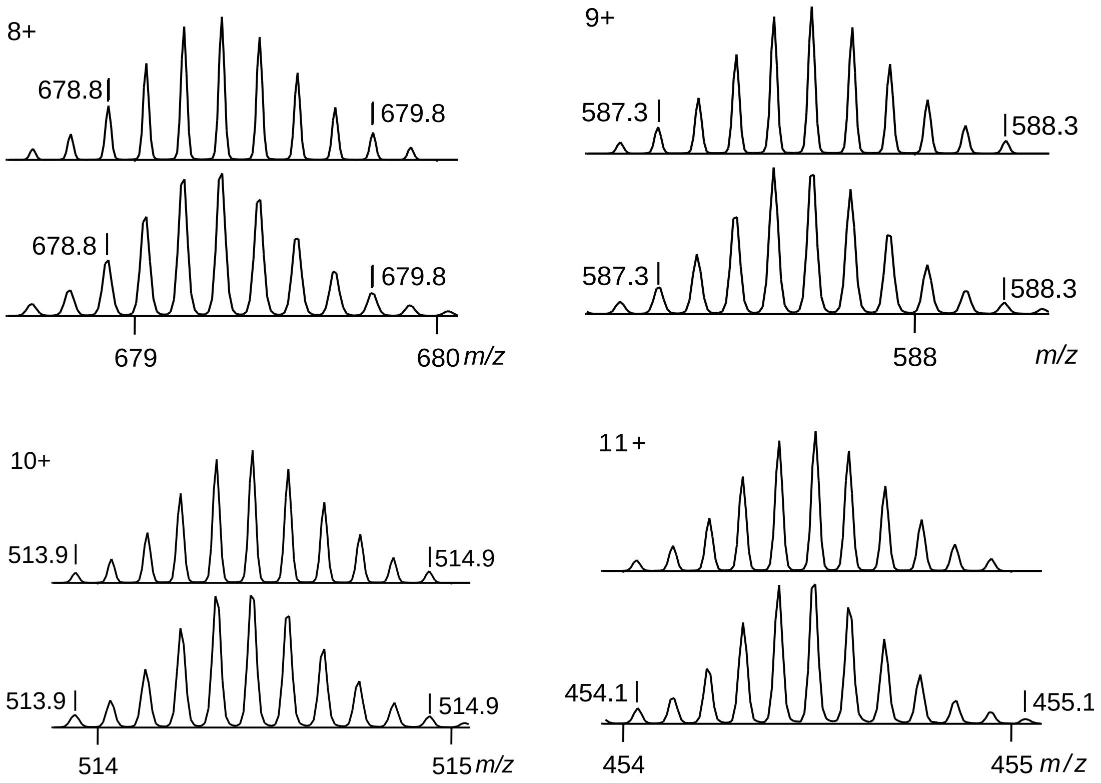

Supplementary Figure 9: Measured (bottom) and calculated (top) isotope patterns for different charge states observed from metallo-bowtie $[Fe_6\boldsymbol{V}_4\boldsymbol{X}]^{12+}$ (PF_6^- as counterion).

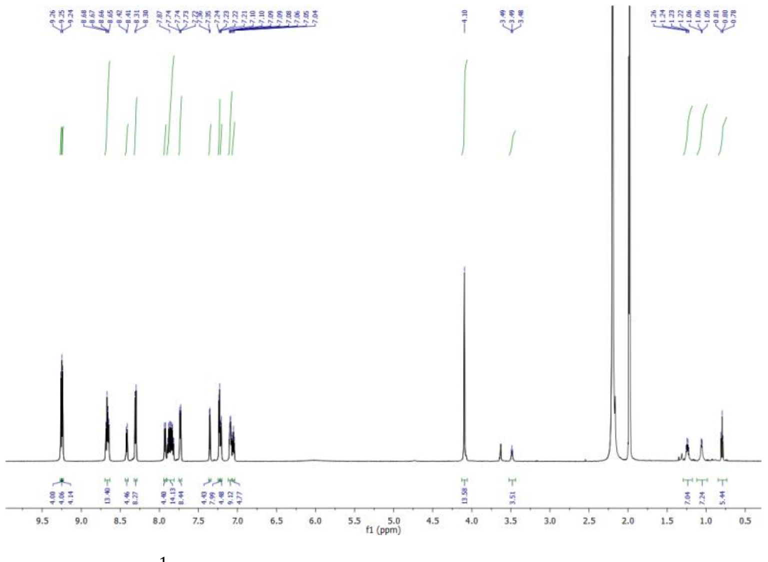

Supplementary Figure 10. ^1H NMR (600 MHz, CD_3CN as solvent) spectrum of metallo-bowtie $[Fe_6\boldsymbol{V}_4\boldsymbol{X}]^{12+}$.

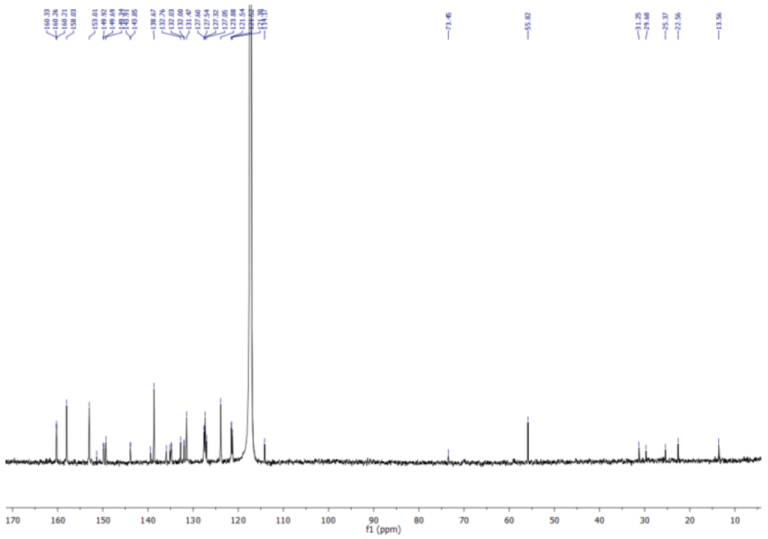

Supplementary Figure 11. ^{13}C NMR (600 MHz, CD_3CN as solvent) spectrum of metallo-bowtie $[Fe_6\boldsymbol{V}_4\boldsymbol{X}]^{12+}$.

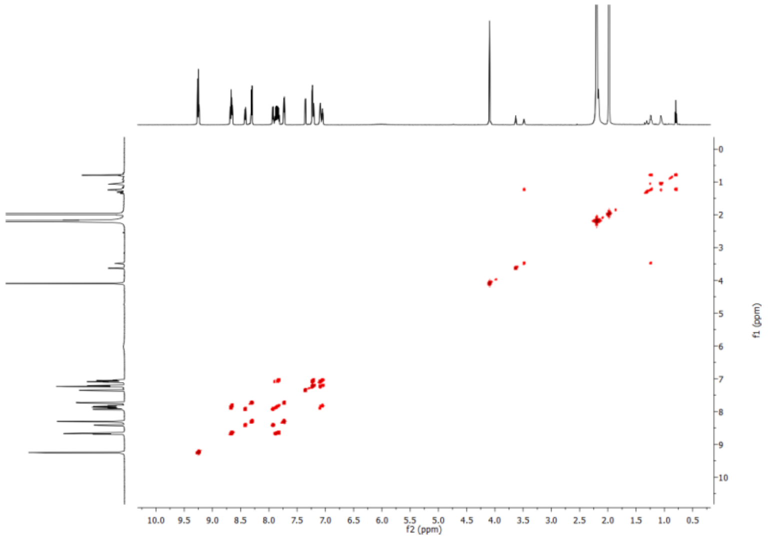

Supplementary Figure 12. 2D COSY NMR (600 MHz, CD_3CN as solvent) spectrum of metallo-bowtie $[Fe_6\boldsymbol{V}_4\boldsymbol{X}]^{12+}$ in CD_3CN.

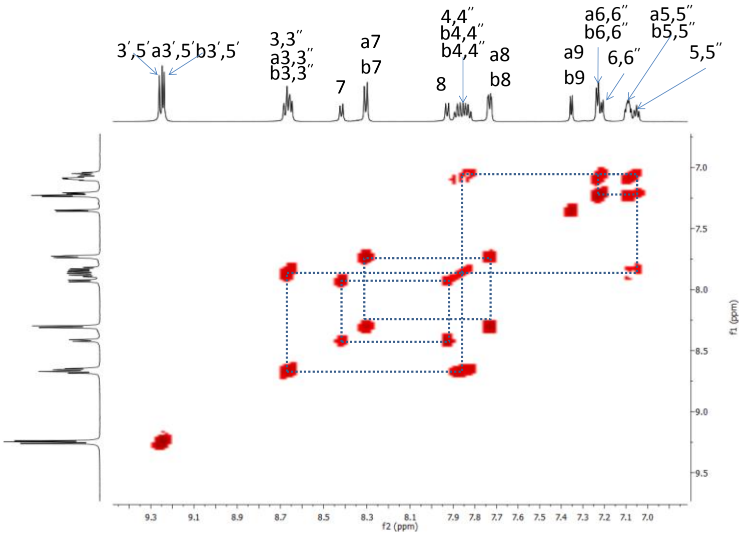

Supplementary Figure 13. 2D COSY NMR (600 MHz, CD_3CN as solvent) spectrum of metallo-bowtie $[Fe_6\boldsymbol{V}_4\boldsymbol{X}]^{12+}$ (aromatic region).

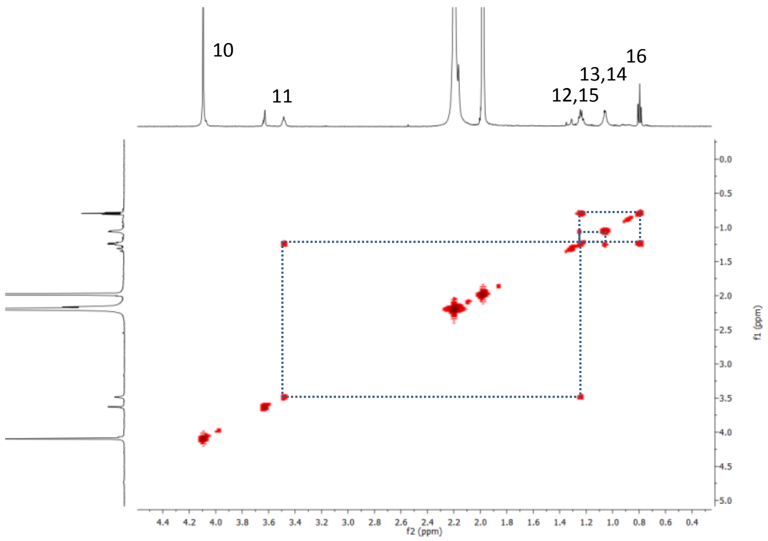

Supplementary Figure 14. 2D COSY NMR (600 MHz, CD_3CN as solvent) spectrum of metallo-bowtie $[Fe_6\boldsymbol{V}_4\boldsymbol{X}]^{12+}$ (aliphatic region).

2.6.3. Tri-metallotriangle $[Fe_9\boldsymbol{V}_5\boldsymbol{X}_2]^{18+}$:

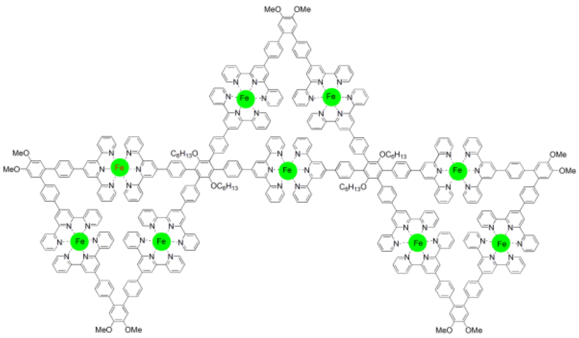

The quantity of this complex is too small to obtain a clear NMR spectra, so only HR-MS has been measured which could confirm the structure. m.p. >320°C; ESI MS (CD_3CN as solvent, *m/z*): 1503.6 $[M-6PF_6]^{6+}$ (calcd *m/z*: 1503.6), 1268.2 $[M-7PF_6]^{7+}$ (calcd *m/z*:

1268.2), 1091.6 [M-8PF$_6$]$^{8+}$ (calcd *m/z*: 1091.6), 954.2 [M-9PF$_6$]$^{9+}$ (calcd *m/z*: 954.2), 844.3 [M-10PF$_6$]$^{10+}$ (calcd *m/z*: 844.3), 754.4 [M-11PF$_6$]$^{11+}$ (calcd *m/z*: 754.4), 679.4 [M-12PF$_6$]$^{12+}$ (calcd *m/z*: 679.4), 615.9[M-13PF$_6$]$^{13+}$ (calcd *m/z*: 615.9) and 561.7 [M-11PF$_6$]$^{14+}$ (calcd *m/z*: 561.7).

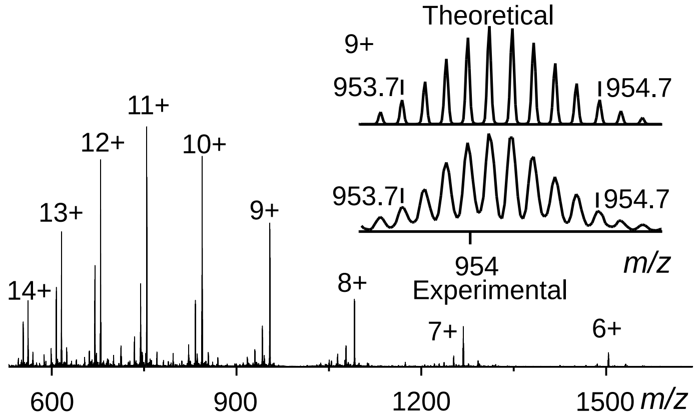

Supplementary Figure 15. ESI-MS of Tri-metallotriangle [Fe$_9$$\boldsymbol{V}_5$$\boldsymbol{X}_2$]$^{18+}$. The charge states of intact assemblies are marked.

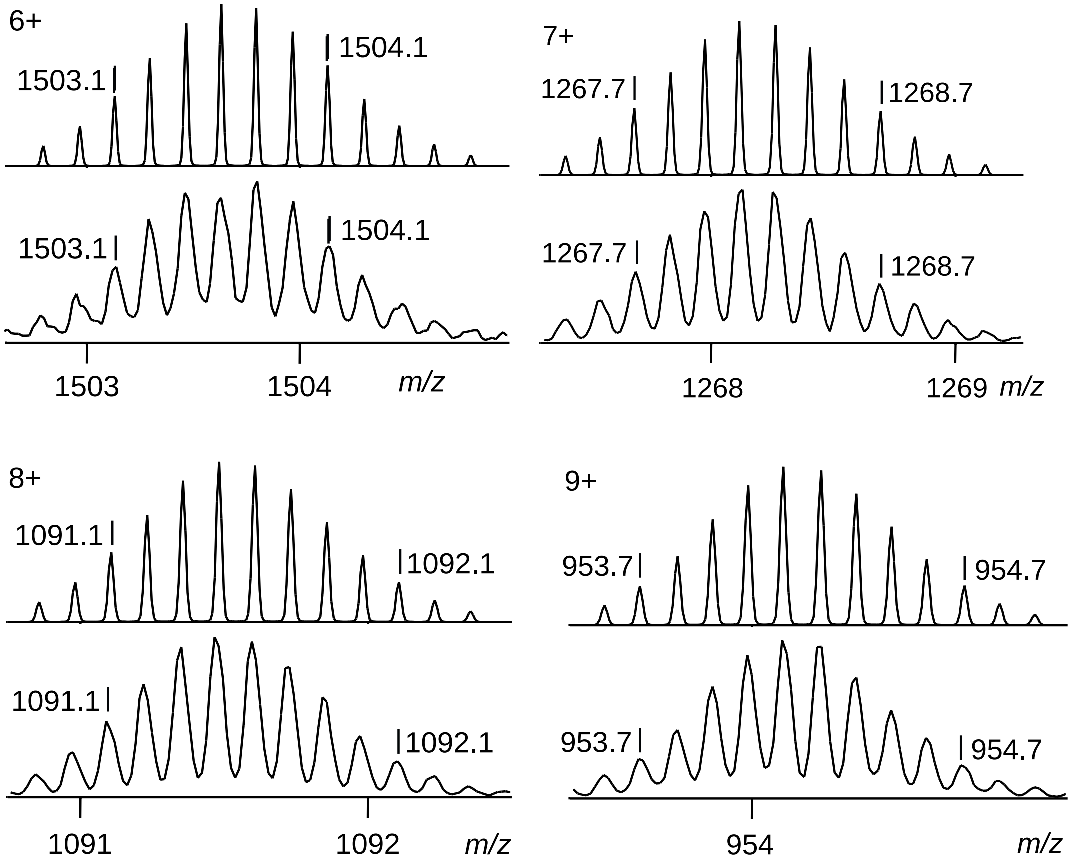

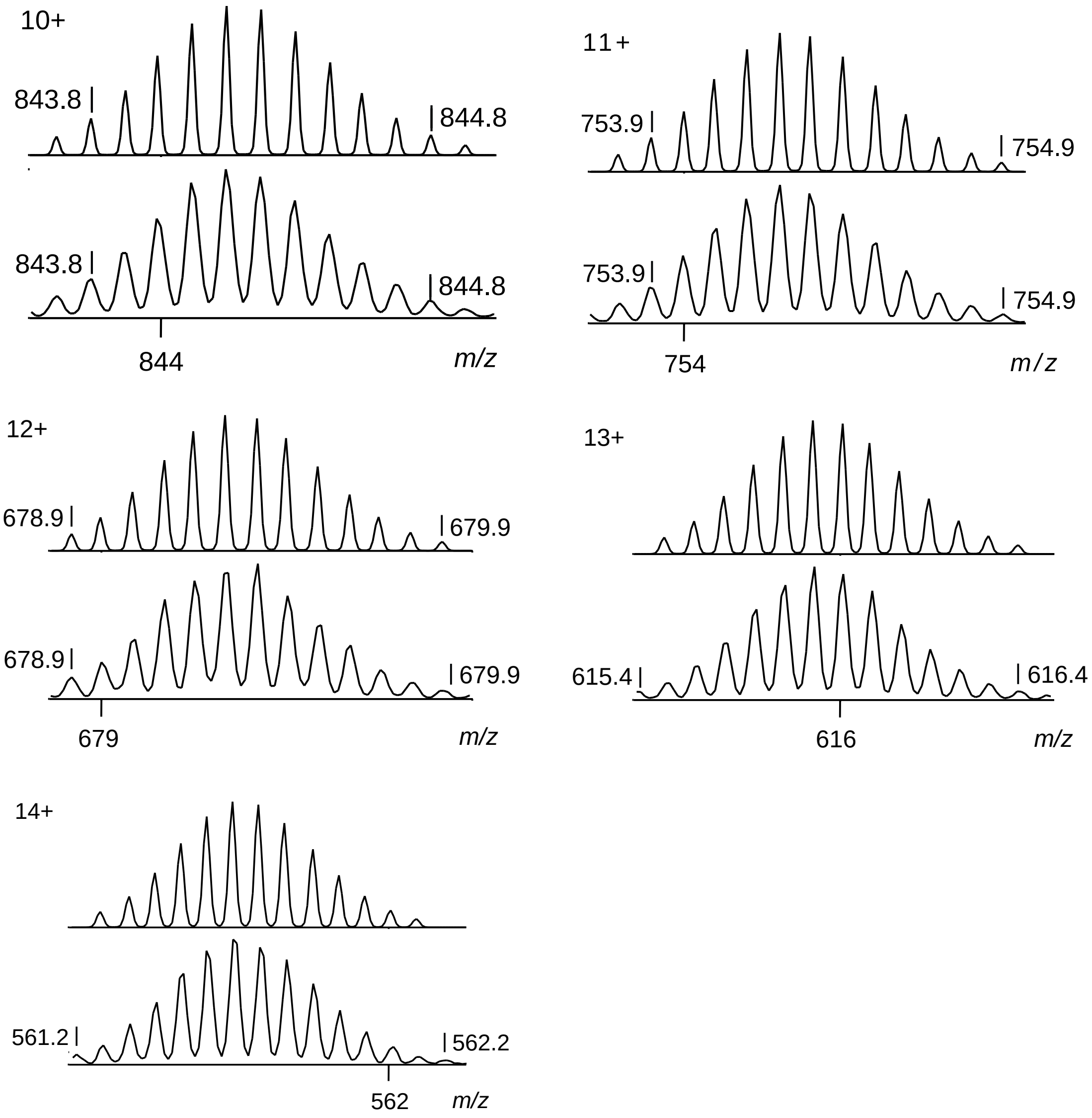

Supplementary Figure 16. Measured (bottom) and calculated (top) isotope patterns for different charge states observed from Tri-metallotriangle $[Fe_9\boldsymbol{V}_5\boldsymbol{X}_2]^{18+}$ (PF_6^- as counterion).

2.7. supramolecular pentagram $[Cd_{10}\boldsymbol{LA}_5]^{30+}$:

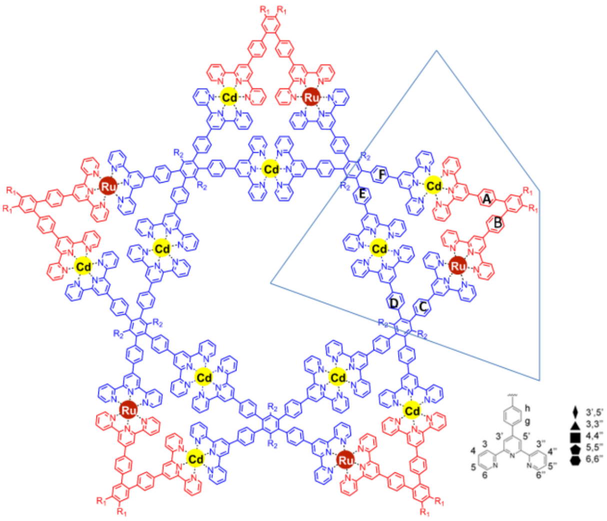

To a solution of ligand ***LA*** (4.86 mg, 2 μmol) in $CHCl_3$/MeOH (10 mL, 1:1 v/v), a solution of $Cd(NO_3)_2$•$6H_2O$ (1.232 mg, 4 μmol) in MeOH (5 mL) was added in drop; then the mixture was stirred at ambient for 8 hours. NH_4PF_6 was added to generate an orange precipitate, which was washed with water and MeOH to obtain a pale reddish product (6.57 mg, 95%). ^{1}H NMR (400 MHz, CD_3CN) δ 9.06 (m, tpy$^{B}H^{3',5'}$, tpy$^{C}H^{3',5'}$, 20H), 8.99(m, tpy$^{A}H^{3',5'}$, tpy$^{D}H^{3',5'}$, tpy$^{E}H^{3',5'}$, tpy$^{F}H^{3',5'}$, 40H)8.80 (m, tpy$^{A}H^{3,3''}$, tpy$^{D}H^{3',3''}$, tpy$^{E}H^{3',3''}$, tpy$^{F}H^{3',3''}$40H), 8.69m, (tpy$^{B}H^{3,3''}$, tpy$^{C}H^{3,3''}$, 20H), 8.16 (m, tpy$^{A}H^{g}$, tpy$^{B}H^{g}$, tpy$^{C}H^{g}$, tpy$^{D}H^{g}$, tpy$^{E}H^{g}$, tpy$^{F}H^{g}$, tpy$^{A}H^{4,4''}$, , tpy$^{D}H^{4,4''}$, tpy$^{E}H^{4,4''}$, tpy$^{F}H^{4,4''}$, 100H), 7.87 (m, tpy$^{B}H^{4,4''}$, tpy$^{C}H^{4,4''}$20H), 7.79 (m, tpy$^{C}H^{h}$, tpy$^{D}H^{h}$, tpy$^{E}H^{h}$, tpy$^{F}H^{h}$,40H), 7.64(m, tpy$^{A}H^{h}$, tpy$^{B}H^{h,}$, tpy$^{B}H^{5,5''}$, tpy$^{C}H^{5,5''}$ 40H), 7.48 (m, tpy$^{A}H^{5,5''}$, tpy$^{D}H^{5,5''}$ tpy$^{E}H^{5,5''}$, tpy$^{F}H^{5,5''}$, tpy$^{A}H^{6,6''}$, tpy$^{D}H^{6,6''}$,60H), 7.30(S, ph^{m,} , 5H), 7.20 (m, ph^{n} 5H), 7.15-7.18(m, tpy$^{B}H^{6,6''}$, tpy$^{C}H^{6,6''}$, tpy$^{E}H^{6,6''}$, tpy$^{F}H^{6,6''}$, 40H) 4.05(m, H^{OMe}, 30H), 3.36-3.20(m, H^{OCH2R}, 20H), 0.89 (m, J = 6.8 Hz, 88H), 0.63 (m, 26H). ESI-MS (CD_3CN as solvent, m/z): 1583.1 $[M-10PF_6]^{10+}$ (calcd *m/z*:1583.1), 1426.0$[M-11PF_6]^{11+}$ (calcd *m/z*:1426.0), 1295.1$[M-12PF_6]^{12+}$(calcd *m/z*:1295.1), 1184.40 $[M-13PF_6]^{13+}$(calcd m/z: 1184.40), 1089.4.$[M-14PF_6]^{14+}$(calcd m/z:1089.4), 1007.19 $[M-15PF_6]^{15+}$(calcd m/z: 1007.19), 935.25$[M-16PF_6]^{16+}$(calcd m/z: 935.25), 871.60 $[M-17PF_6]^{17+}$ (calcd m/z: 871.60), 815.1$[M-18PF_6]^{18+}$ (calcd m/z: 815.1), 764.45 $[M-19PF_6]^{19+}$ (calcd m/z: 764.5), 719.0 $[M-20PF_6]^{20+}$ (calcd m/z: 719.0), 677.9 $[M-21PF_6]^{21+}$ (calcd m/z: 677.9).

2.8. supramolecular pentagram [Fe_{10}(*LA*)$_5$]$^{30+}$

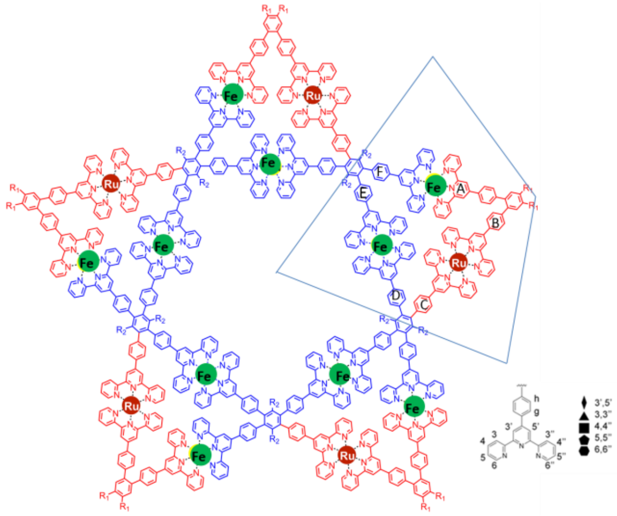

To a solution of ligand ***LA***(4.86 mg, 2 μmol) and $FeCl_2$•$4H_2O$ (0.796 mg, 4 μmol) in glycol (10 mL), the mixture was heated to 145℃ for 12 hours. After cooling to ambient, 50mL MeOH was added to the solution and stirred for 2 hours. then excess NH_4PF_6 was added to cause a purple precipitate, after washed with water and MeOH and dried in vacuum, a reddish solid was collected (6.38 mg, 95%). ^{1}H NMR (500 MHz, CD_3CN) δ 9.30-9.09 (m, tpy^A$H^{3',5'}$, tpy^B$H^{3',5'}$, tpy^C$H^{3',5'}$, tpy^D$H^{3',5'}$, tpy^E$H^{3',5'}$, tpy^F$H^{3',5'}$, 60H), 8.78 – 8.58 (m, tpy^A$H^{3,3''}$, tpy^B$H^{3,3''}$, tpy^C$H^{3,3''}$, tpy^D$H^{3,3''}$, tpy^E$H^{3,3''}$, tpy^F$H^{3,3'}$, 60H), 8.25 (m, tpy^AH^g, tpy^BH^g , tpy^CH^g , tpy^DH^g, tpy^EH^g , tpy^FH^g, 72H), 8.05 – 7.75 (m, tpy^AH^h , tpy^BH^h tpy^CH^h, tpy^DH^h, tpy^EH^h, tpy^FH^h,72H), 7.67 (m, tpy^A$H^{4,4''}$, tpy^D$H^{4,4''}$, tpy^E$H^{4,4''}$, tpy^F$H^{4,4''}$, 48H), 7.50 – 7.36 (m, tpy^B$H^{4,4''}$, tpy^C$H^{4,4''}$ 24H), 7.31 (m, tpy^B$H^{5,5''}$, tpy^C$H^{5,5''}$ 20H), 7.26 – 6.92 (m, ph^m , ph^n, tpy^A$H^{6,6''}$, tpy^B$H^{6,6''}$, tpy^C$H^{6,6'}$, tpy^D$H^{6,6''}$, tpy^E$H^{6,6''}$, tpy^F$H^{6,6'''}$, tpy^A$H^{5,5''}$, tpy^D$H^{5,5''}$, tpy^E$H^{5,5''}$, tpy^F$H^{5,5''}$ 70H), 4.06 (s, H^{OMe} 32H), 3.57 (s, H^{OCH2R} 25H). ESI-MS (CD_3CN as solvent, m/z): 1712.4 [M-9PF_6⏋$^{9+}$ (calcd m/z: 1712.4), 1526.6 [M-10PF_6⏋$^{10+}$ (calcd m/z: 1526.6), 1374.4[M-11PF_6⏋$^{11+}$ (calcd m/z: 1374.4), 1248.0 [M-12PF_6⏋$^{12+}$ (calcd m/z: 1248.0), 1140.8 [M-13PF6⏋$^{13+}$ (calcd m/z: 1140.8), 1049.1 [M-14PF_6⏋$^{14+}$ (calcd m/z:1049.1), 969.5 [M-15PF_6⏋$^{15+}$ (calcd m/z: 969.5), 899.9 [M-16PF_6⏋$^{16+}$ (calcd m/z: 899.9), 838.4[M-17PF_6⏋$^{17+}$ (calcd m/z: 838.4), 784.0 [M-18PF_6⏋ $^{18+}$ (calcd m/z: 784.0), 734.8 [M-19PF_6⏋$^{19+}$ (calcd m/z: 734.8), 690.8 [M-20PF_6⏋$^{20+}$ (calcd m/z: 690.8), 651.0[M-21PF_6⏋$^{21+}$ (calcd m/z: 651.0), 614.8 [M-22PF_6⏋ $^{22+}$ (calcd m/z: 614.8).

2.9. supramolecular hexagram $[Cd_{12}\boldsymbol{V}_3(\boldsymbol{LB})_3]^{36+}$

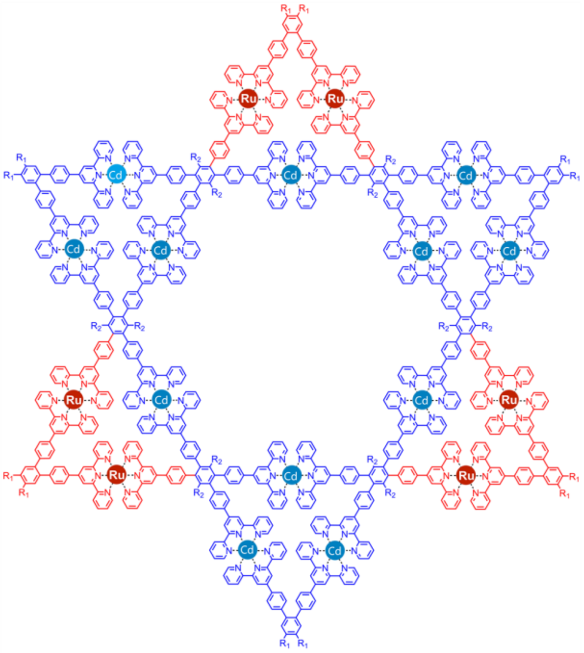

To a solution of ligand ***LB*** (8.90 mg, 2 μmol) and ***V*** (1.51 mg, 2 μmol) in $CHCl_3$/MeOH (10 mL, 1:1 v/v), a methanolic solution of $Cd(NO_3)_2$•$6H_2O$ (2.464 mg, 8 μmol) was added dropwise; then the mixture was stirred at ambient for 8 hours. Excess amount of NH_4PF_6 was added to generate a pale reddish precipitate, after washing water and MeOH and dried under vacuum to obtain reddish solid 14 mg. NMR and Mass spectra indicated there are two compounds (see Figure S50 and S51), including a metallo-triangle and a target Star-Of-David, which could not be separated by a conventional column chromatography due to the week coordination of <tpy-Cd^{2+}-tpy>. The ESI-MS of Star-Of-David are list as following: ESI-MS(CD_3CN as solvent, m/z): 1832.1 $[M\text{-}11PF_6]^{11+}$ (calcd m/z: 1832.1), 1667.9 $[M\text{-}12PF_6]^{12+}$ (calcd m/z: 1667.9), 1528.3 $[M\text{-}13PF_6]^{13+}$ (calcd m/z: 1528.3), 1408.6 $[M\text{-}14PF_6]^{14+}$ (calcd m/z:1408.6), 1305.1$[M\text{-}15PF_6]^{15+}$ (calcd m/z: 1305.1), 1214.4 $[M\text{-}16PF_6]^{16+}$ (calcd m/z: 1214.4), 1134.5$[M\text{-}17PF_6]^{17+}$ (calcd m/z: 1134.5), 1061.4$[M\text{-}18PF_6]^{18+}$ (calcd m/z: 1061.4), 999.7 $[M\text{-}19PF_6]^{19+}$ (calcd m/z: 999.7) and 942.4 $[M\text{-}19PF_6]^{20+}$ (calcd m/z: 942.4).

2.10. supramolecular hexagram $[Fe_{12}\boldsymbol{V}_3(\boldsymbol{LB})_3]^{36+}$

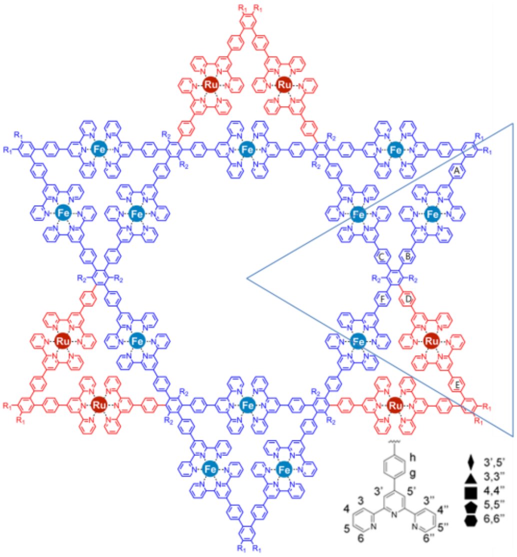

To a solution of ligand ***LB*** (8.90 mg, 2 μmol) and ***V*** (1.51 mg, 2 μmol) in glycol (10 mL, 1:1 v/v), a solution of $FeCl_2$•$4H_2O$ (1.584 mg, 8 μmol) in glycol (5 mL) was added dropwise. The mixture was heated to 145℃ for 12 hours. After cooling down to ambient, 50mL MeOH was added and stirred for another 2 hours, adding excess amount of NH_4PF_6 to produce a purple reddish precipitate.

A short column chromatography was used for isolated the trace amount of triangle byproduct $(Fe_3\boldsymbol{V}_3)^{6+}$. The isolated Star of David was washed with water and MeOH to generate a reddish product 13.20 mg (92%). ^{1}H NMR (500 MHz, CD_3CN) δ 9.2-9.4 (m, 48H, $tpy^{A}H^{3',5'}$, $tpy^{B}H^{3',5'}$, $tpy^{C}H^{3',5'}$, $tpy^{D}H^{3',5'}$), 9.05-9.15 (m, $tpy^{D}H^{3',5'}$, $tpy^{E}H^{3',5'}$, 24H), 8.68-8.80 (m, 72H, $tpy^{A}H^{3,3''}$, $tpy^{B}H^{3,3''}$, $tpy^{C}H^{3,3''}$, $tpy^{D}H^{3,3''}$, $tpy^{E}H^{3,3''}$, $tpy^{F}H^{3,3'}$), 8.15-8.45 (m, $tpy^{A}H^{g}$, $tpy^{B}H^{g}$, $tpy^{C}H^{g}$, $tpy^{D}H^{g}$, $tpy^{E}H^{g}$, 72H), 7.85-8.05 (m, $tpy^{A}H^{h}$, $tpy^{B}H^{g}$, $tpy^{C}H^{h}$, $tpy^{C}H^{h}$, $tpy^{D}H^{h}$, $tpy^{E}H^{h}$, $tpy^{F}H^{h}$, $tpy^{D}H^{4,4''}$, $tpy^{E}H^{4,4''}$, 96H), 7.60-7.70 (m, $tpy^{A}H^{4,4''}$, $tpy^{B}H^{4,4''}$, $tpy^{C}H^{4,4''}$, $tpy^{F}H^{4,4''}$, 48H), 7.32-7.55(m, $tpy^{A}H^{5,5''}$, $tpy^{B}H^{5,5''}$ $tpy^{C}H^{5,5''}$, $tpy^{D}H^{5,5''}$ $tpy^{E}H^{5,5''}$, $tpy^{F}H^{5,5''}$ 72H), 7.05-7.19 (m, 84H), 7.05 (m, ph^{m} , ph^{n}, $tpy^{A}H^{6,6''}$, $tpy^{B}H^{6,6''}$, $tpy^{C}H^{6,6'}$, $tpy^{D}H^{6,6''}$, $tpy^{E}H^{6,6''}$, $tpy^{F}H^{6,6'''}$, $tpy^{A}H^{5,5''}$, $tpy^{D}H^{5,5''}$, $tpy^{E}H^{5,5''}$, $tpy^{F}H^{5,5''}$ 84H), 4.06 (m, 36H, -OMe), 3.44 (m, 24H, $-OCH_2-$), 0.92 (m,78H –Alkyl-H), 0.53-0.68 (m, Alkyl-H, 154H). ESI-MS (CD_3CN as solvent, m/z): 1771.1$[M-11PF_6]^{11+}$, (calcd m/z: 1771.1), 1671.4$[M-12PF_6]^{12+}$ (calcd m/z: 1671.4), 1476.2 $[M-13PF_6]^{13+}$ (calcd m/z:1476.2), 1360.2 $[M-14PF_6]^{14+}$ (calcd m/z: 1360.2), 1259.9 $[M-15PF_6]^{15+}$ (calcd m/z: 1259.9), 1171.8 $[M-16PF_6]^{16+}$ (calcd m/z: 1171.8), 1094.70$[M-17PF_6]^{17+}$, (calcd m/z: 1094.70), 1025.71 $[M-18PF_6]^{18+}$ (calcd m/z:1025.71), 963.41 $[M-19PF_6]^{19+}$ (calcd m/z: 963.98), 908.67 $[M-20PF_6]^{20+}$ (calcd m/z: 908.67), 858.45 $[M-21PF_6]^{21+}$ (calcd m/z: 858.45), 812.7 $[M-22PF_6]^{22+}$ (calcd m/z: 1812.7), 771.3 $[M-23PF_6]^{23+}$ (calcd m/z: 771.3), 733.1 $[M-24PF_6]^{24+}$ (calcd m/z: 733.1).

3. HR-MS, NMR and UV-*vis* spectrum of ligands and complexes

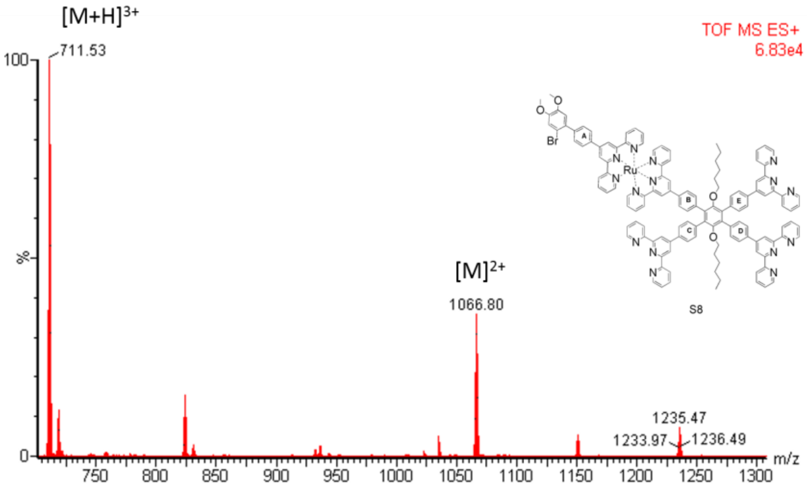

Supplementary Figure 17: The HR-MS spectrum of compound ***L-Br*** in CH_3CN.

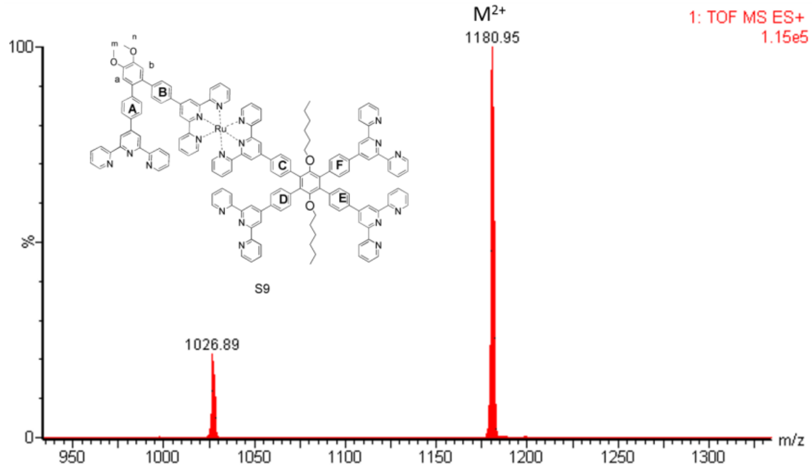

Supplementary Figure 18: The HR-MS spectrum of compound ***LA*** in CH_3CN.

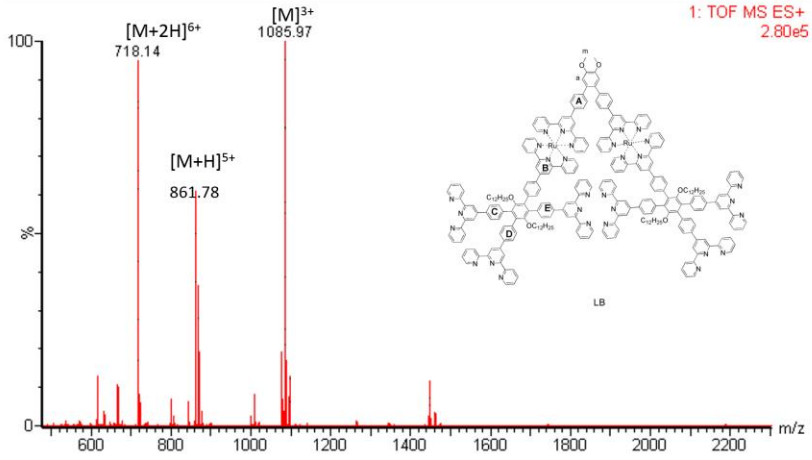

Supplementary Figure 19: The HR-MS spectrum of compound ***LB*** in CH_3CN.

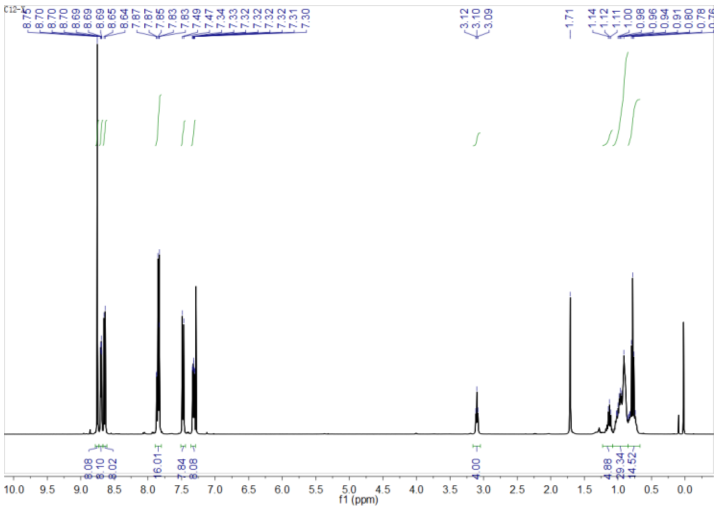

Supplementary Figure 20: the ^{1}H NMR spectrum of ***X-C12*** (500 MHz, $CDCl_3$ as solvent)

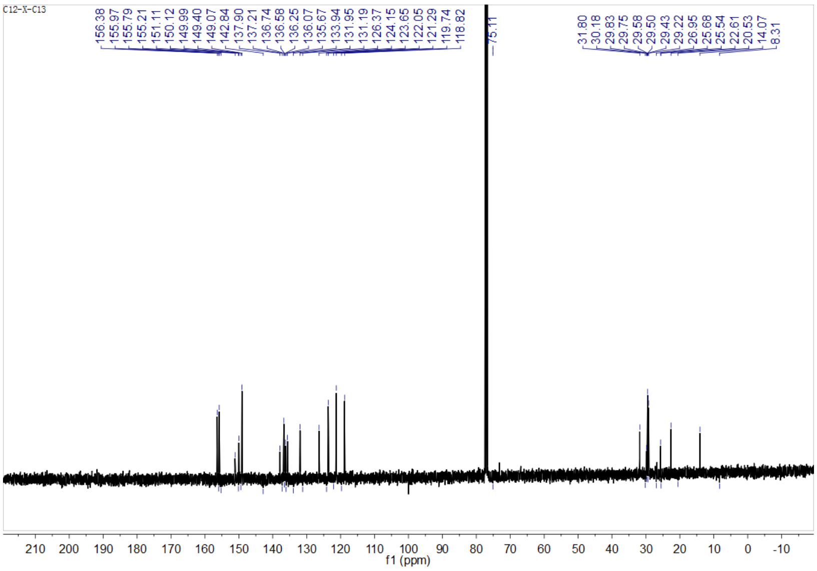

Supplementary Figure 21: the ^{13}C NMR spectrum (125 MHz, $CDCl_3$ as solvent) of ***X-C12***

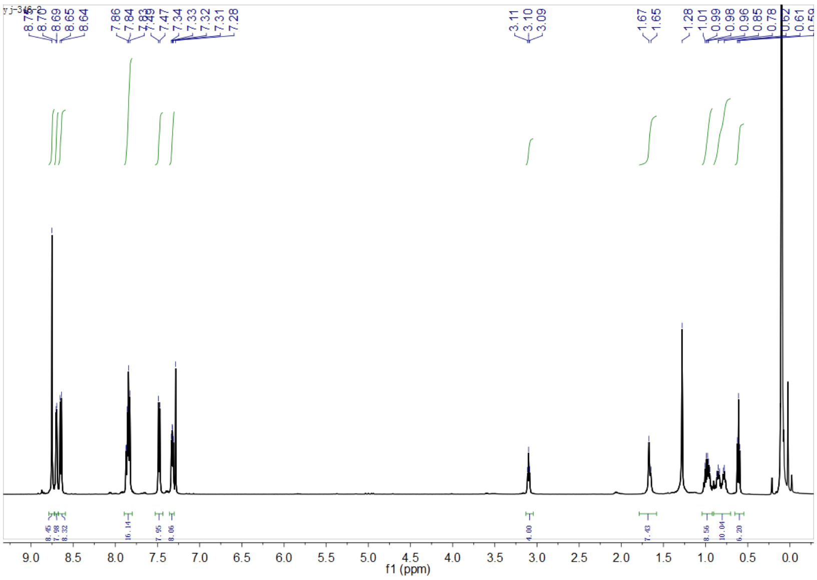

Supplementary Figure 22: the ^{1}H NMR spectrum of ***X-C6***. (500 MHz, $CDCl_3$ as solvent)

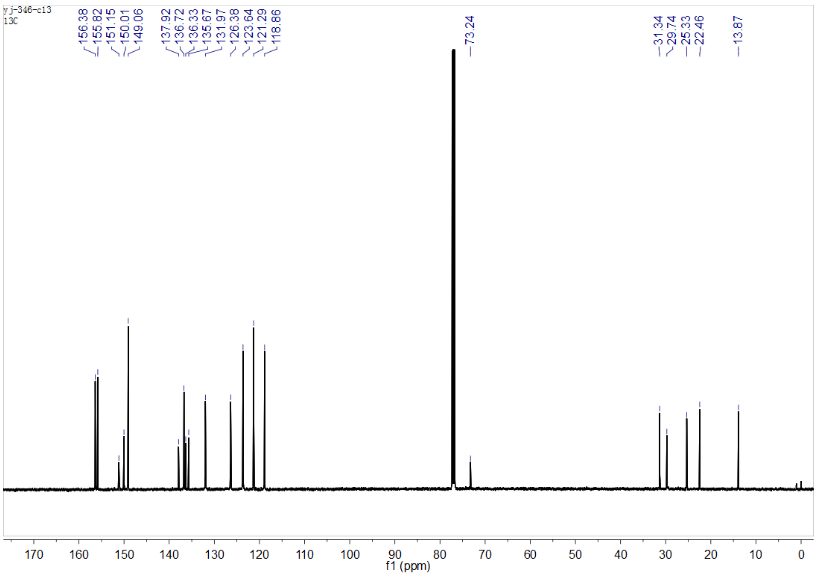

Supplementary Figure 23: the ^{13}C NMR spectrum of ***X-C6*** (125 MHz, $CDCl_3$ as solvent)

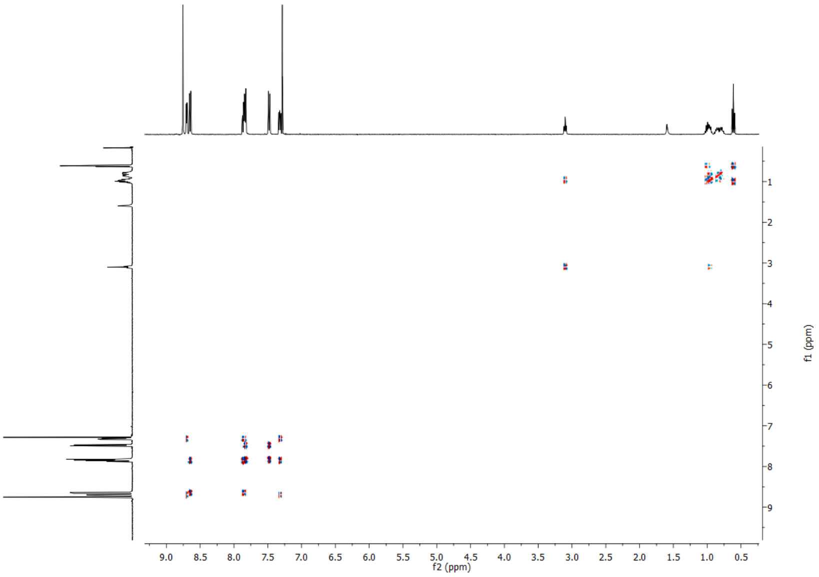

Supplementary Figure 24: the 2D COSY spectrum of Ligand ***X-C6*** (400 MHz, $CDCl_3$ as solvent)

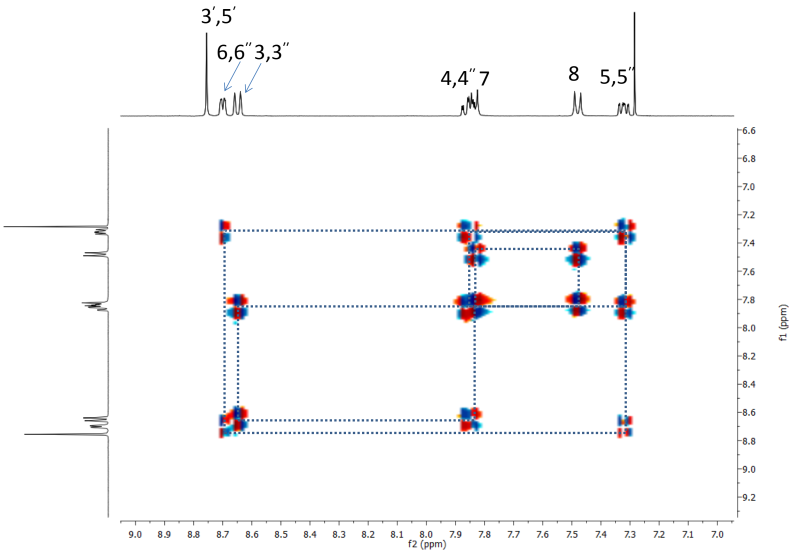

Supplementary Figure 25: the COSY spectrum of Ligand ***X-C6***. (400 MHz, $CDCl_3$ as solvent, aromatic region).

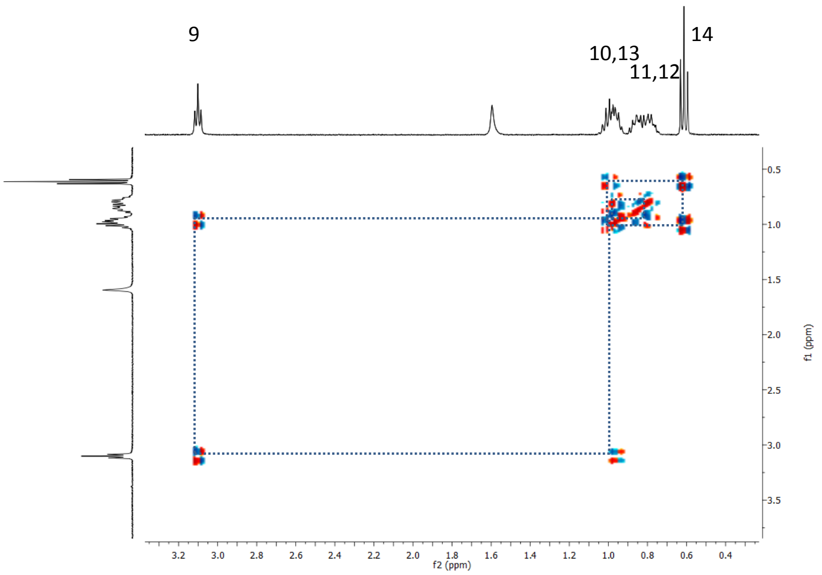

Supplementary Figure 26: the 2D COSY spectrum of Ligand ***X-C6*** (400 MHz. $CDCl_3$ as solvent, aliphatic region)

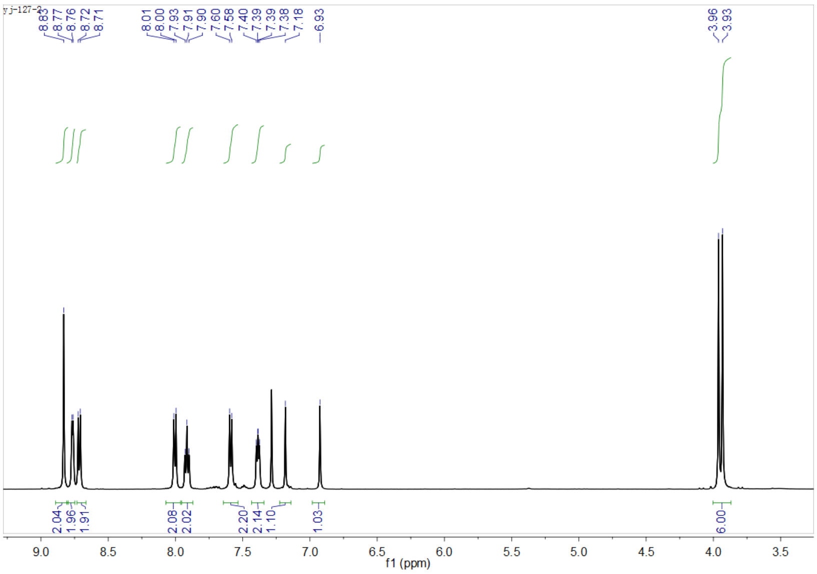

Supplementary Figure 27: the ^{1}H NMR spectrumof **monobromoterpyridine** (400 MHz, $CDCl_3$ as solvent)

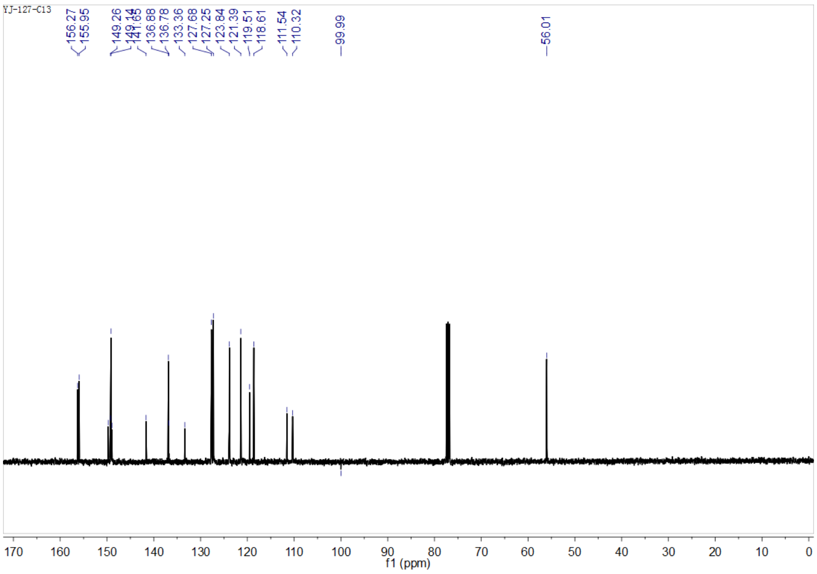

Supplementary Figure 28: the ^{13}C NMR spectrumof **monobromoterpyridine** (125 MHz, $CDCl_3$ as solvent).

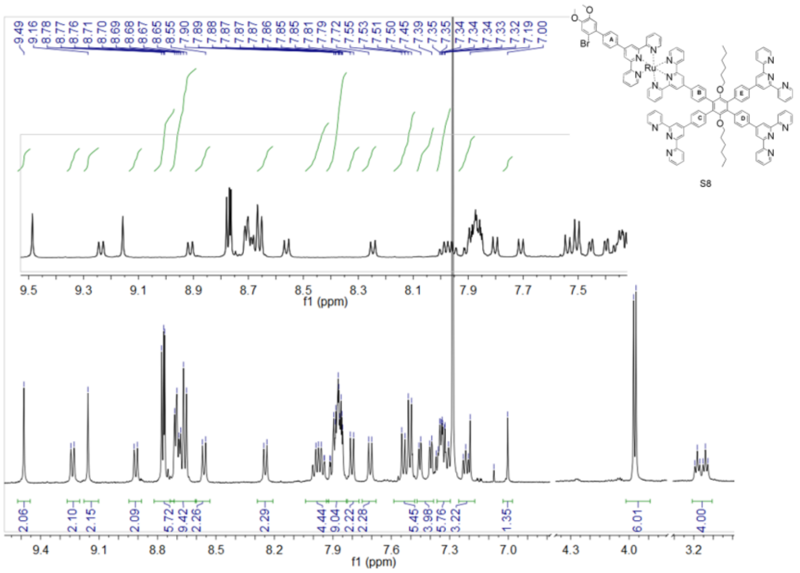

Supplementary Figure 29: the ^{1}H NMR spectrum of ***L-Br***. (aromatic region, 500 MHz, $CDCl_3$ as solvent)

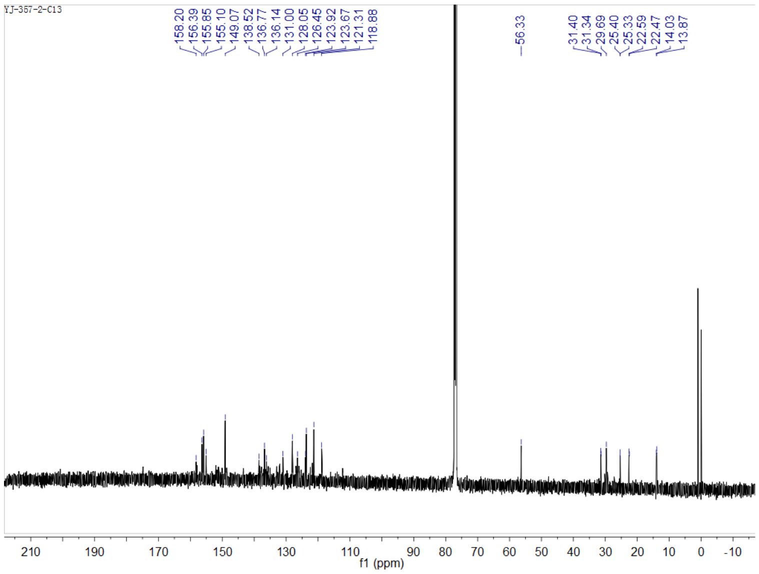

Supplementary Figure 30: the ^{13}C NMR spectrum of ***L-Br*** (125 MHz, $CDCl_3$ as solvent).

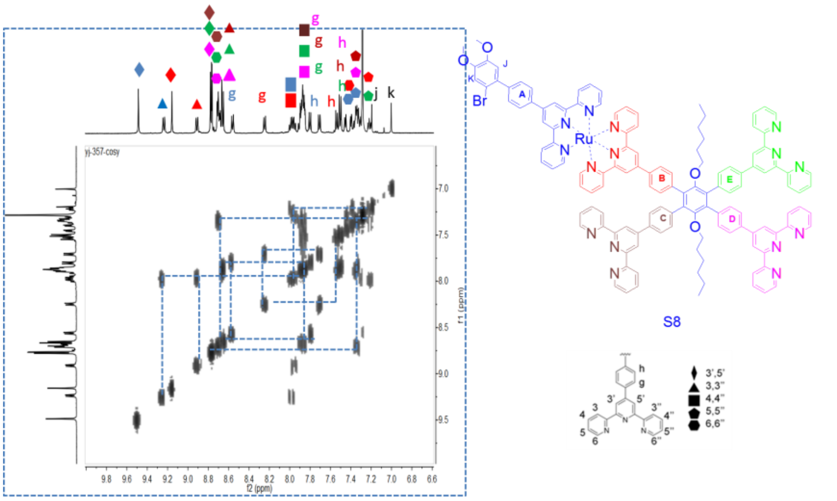

Supplementary Figure 31: the COSY spectrum of ***L-Br*** (aromatic region, 500 MHz, $CDCl_3$ as solvent)

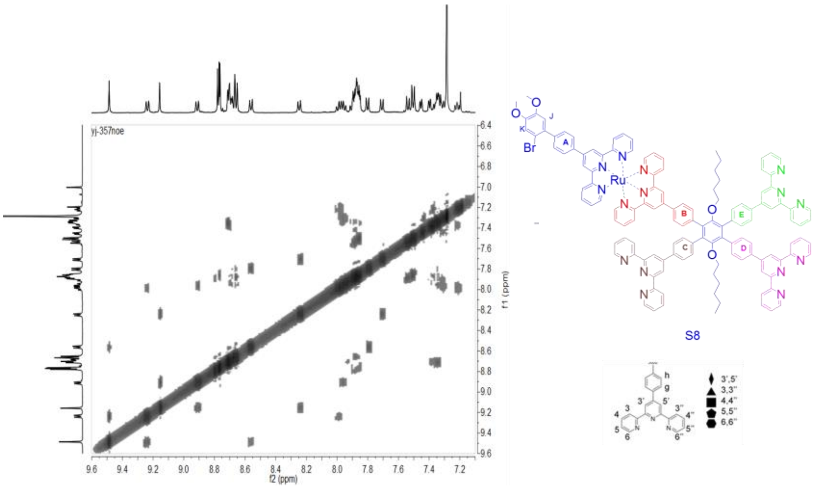

Supplementary Figure 32: the NOESY spectrum of ***L-Br*** (aromatic region, 500 MHz, $CDCl_3$ as solvent).

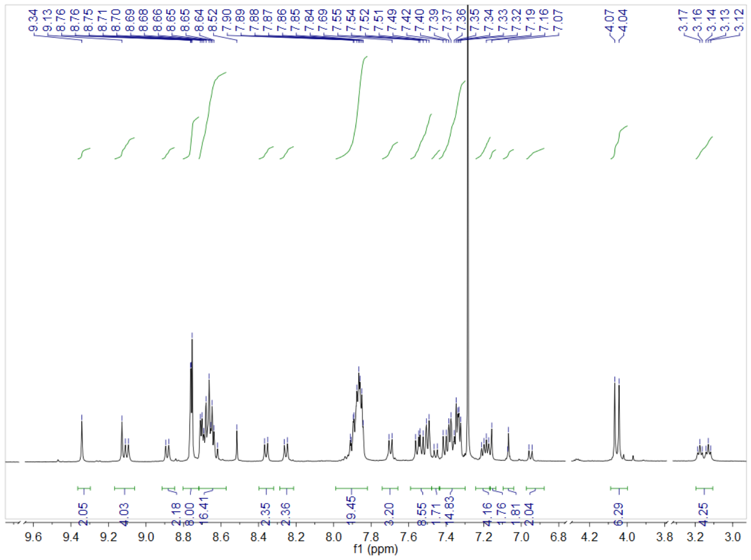

Supplementary Figure 33: the ^{1}H NMR spectrum of ***LA*** (500 MHz, $CDCl_3$ as solvent).

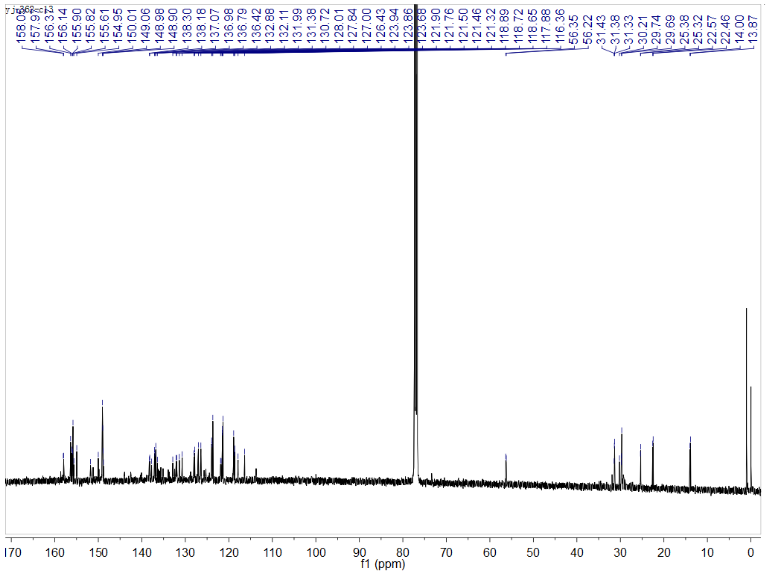

Supplementary Figure 34: the ^{13}C NMR spectrum of **LA**. (125 MHz, $CDCl_3$ as solvent)

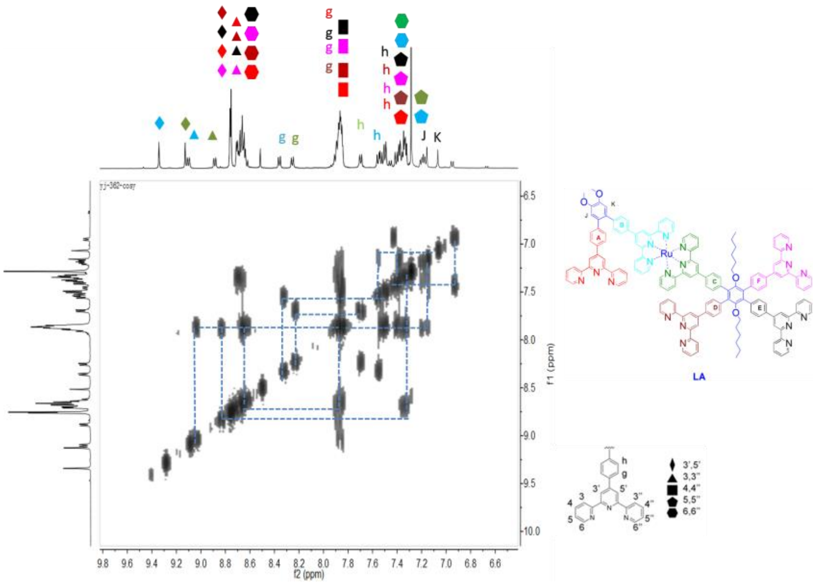

Supplementary Figure 35: the COSY spectrum of **LA.** (aromatic region, 500 MHz, $CDCl_3$ as solvent)

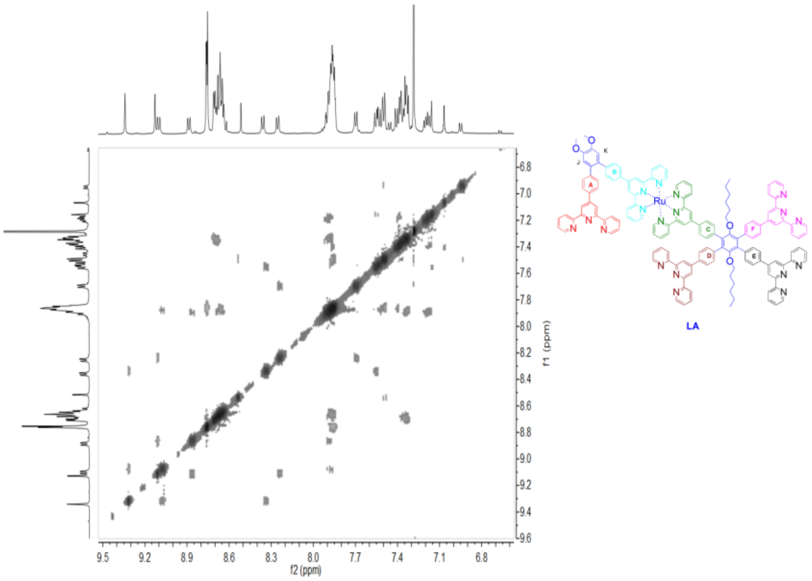

Supplementary Figure 36: the NOESY spectrum of ***LA*** (aromatic region, 500 MHz, $CDCl_3$ as solvent).

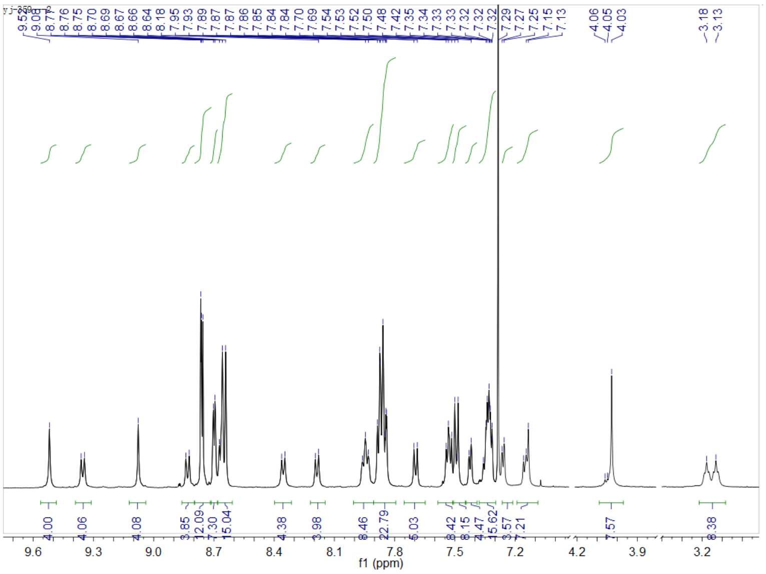

Supplementary Figure 37: the ^{1}H NMR spectrum of ***LB*** (500 MHz, $CDCl_3$ as solvent).

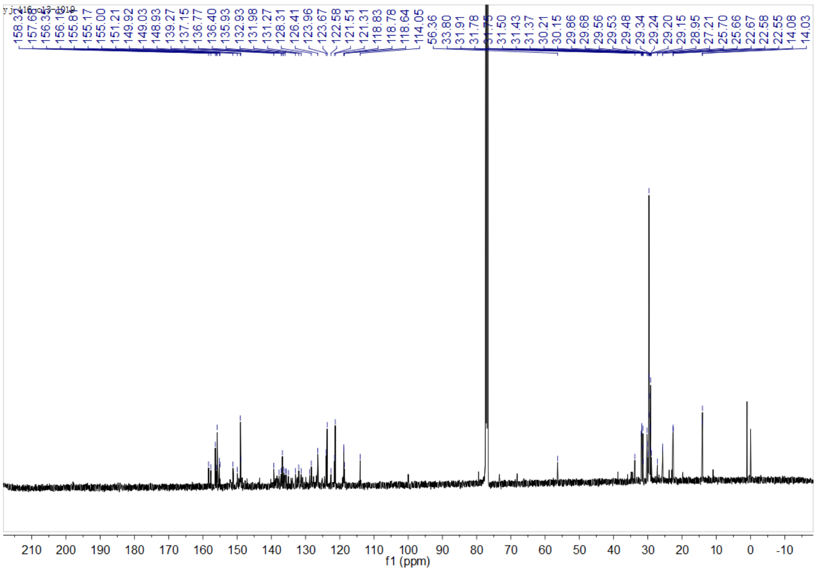

Supplementary Figure 38: the ^{13}C NMR spectrum of ***LB***. (125 MHz, $CDCl_3$ as solvent)

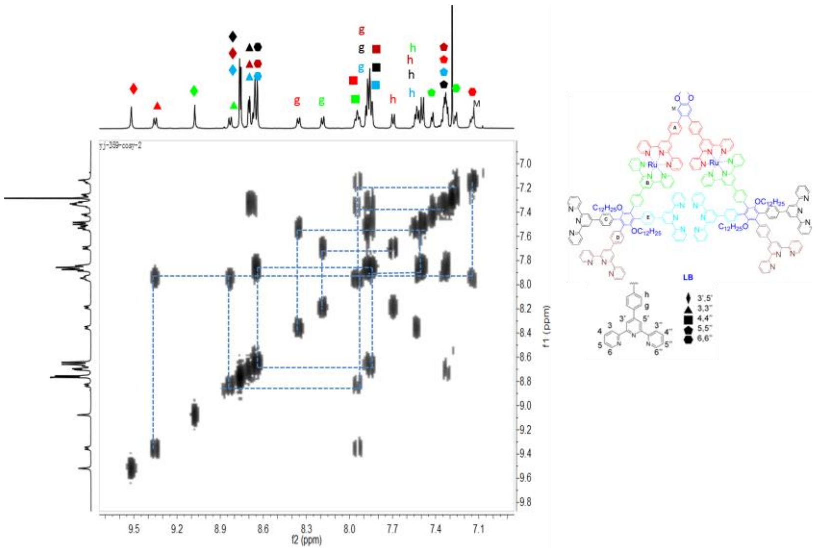

Supplementary Figure 39: the COSY spectrum of ***LB***. (500 MHz, $CDCl_3$ as solvent)

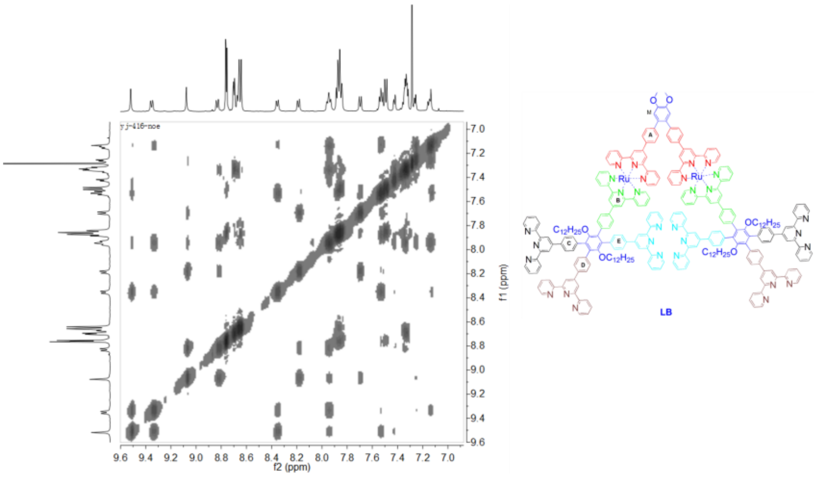

Supplementary Figure 40: the NOESY spectrum of ***LB***. (aromatic region, 500 MHz, $CDCl_3$ as solvent)

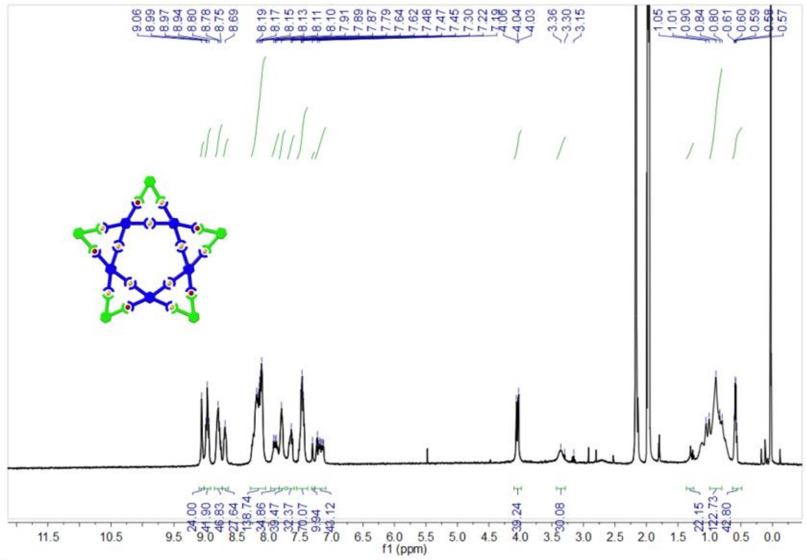

Supplementary Figure 41: the ^{1}H NMR spectrum of $[Cd_{10}\boldsymbol{LA}_5]^{30+}$ •$30PF_6^-$ (500 MHZ, CD_3CN as solvent).

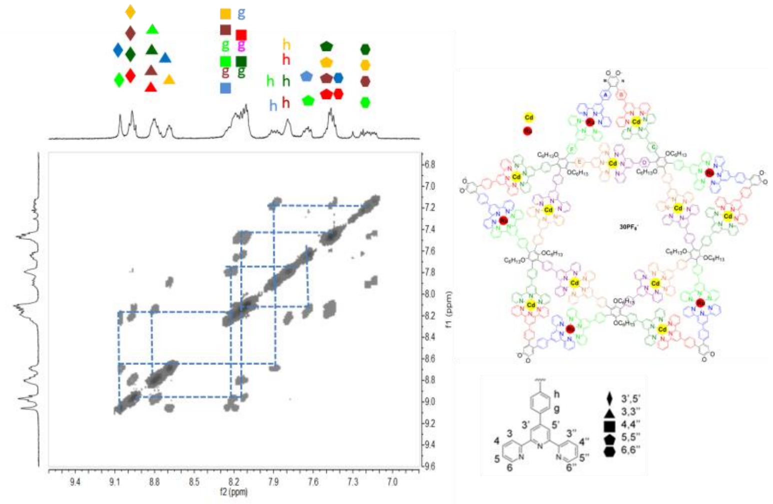

Supplementary Figure 42: the NOESY spectrum of $[\boldsymbol{Cd_{10}LA_5}]^{\mathbf{30+}}$ • $30PF_6^-$ (aromatic region, 500 MHz, CD_3CN as solvent).

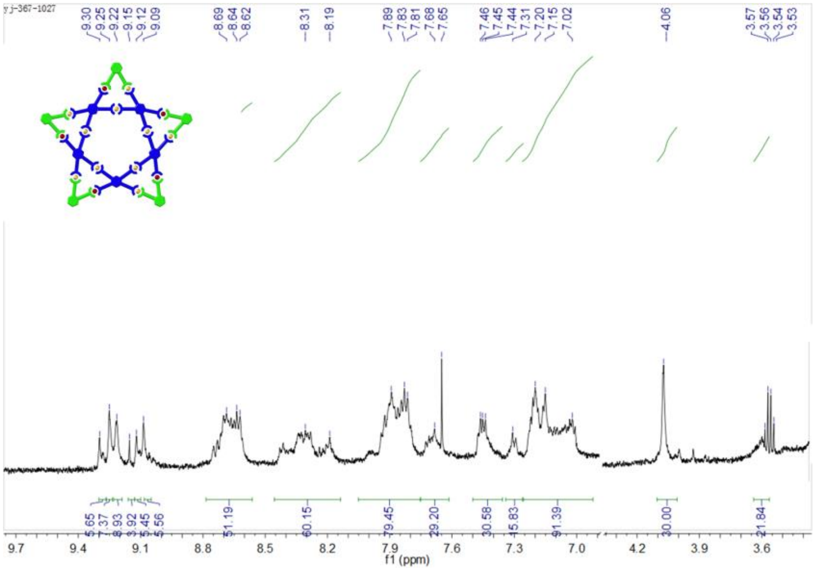

Supplementary Figure 43: the ^{1}H NMR spectrum of $[Fe_{10}\boldsymbol{LA}_5]^{30+}$ • $30PF_6^-$ (500 MHz, CD_3CN as solvent)

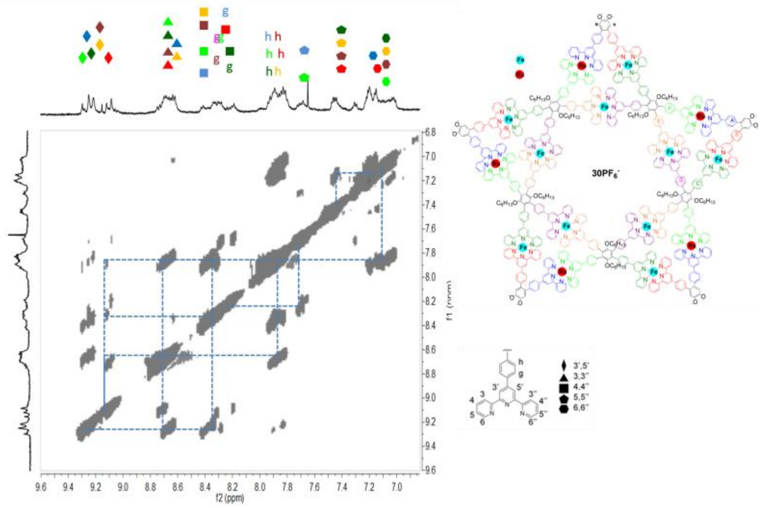

Supplementary Figure 44: the NOESY spectrum of $[Fe_{10}\boldsymbol{LA}_5]^{30+}$ • $30PF_6^-$ (aromatic region, 500 MHz, CD_3CN as solvent)

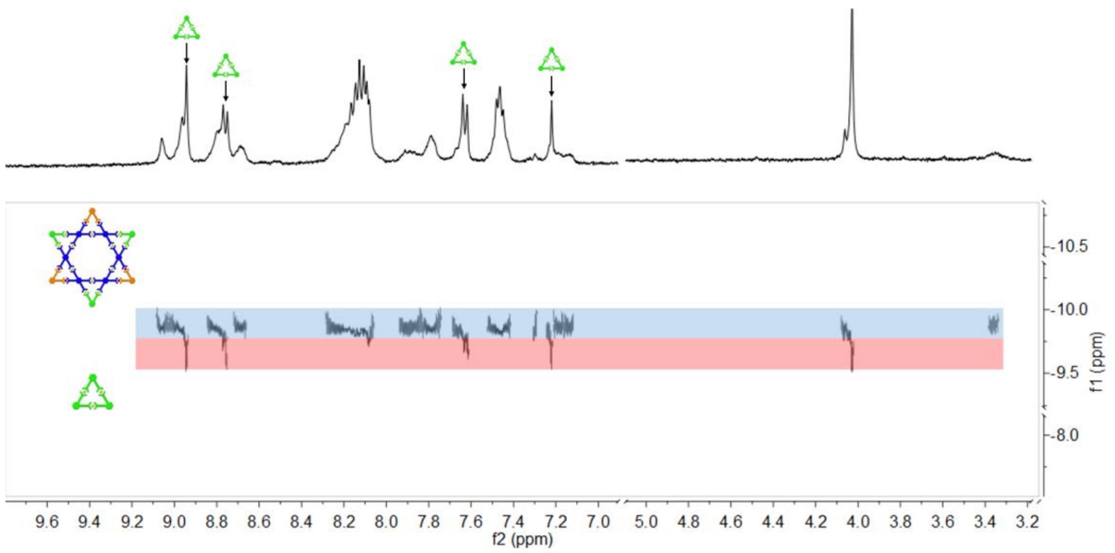

Supplementary Figure 45: the ^{1}H NMR and DOSY spectrum (500 MHz, CD_3CN as solvent) of $[Cd_{12}\boldsymbol{V}_3(\boldsymbol{LB})_3]^{36+}$ • $36PF_6^-$ and $[Cd_3\boldsymbol{V}_3]^{6+}X$ • $6PF_6^-$ assembled directly with Cd^{2+}, ***V*** and ***LB*** which containing small triangle and Star-Of-David.

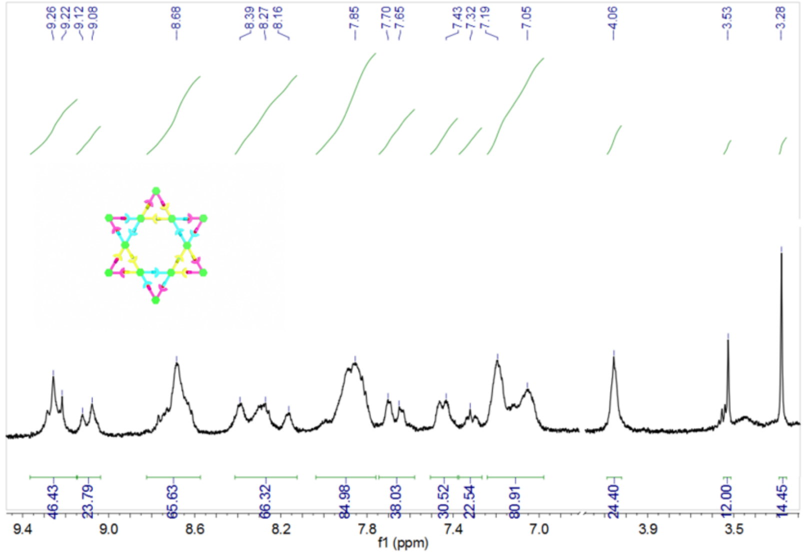

Supplementary Figure 46: the ^{1}H NMR spectrum of $[Fe_{12}\boldsymbol{V}_3(\boldsymbol{LB})_3]^{36+}$ •$36PF_6^-$ (500MHz, CD_3CN as solvent) assembled with Fe^{2+}, ***V*** and ***LB***.

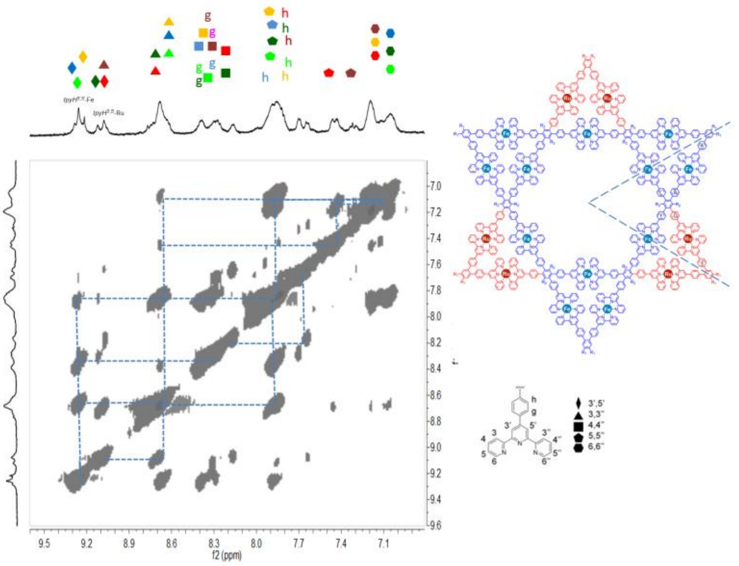

Supplementary Figure 47: the NOESY spectrum of $[Fe_{12}\boldsymbol{V}3(\boldsymbol{LB})_3]^{36+}$ • $36PF_6^-$ (500 MHz, CD_3CN as solvent).

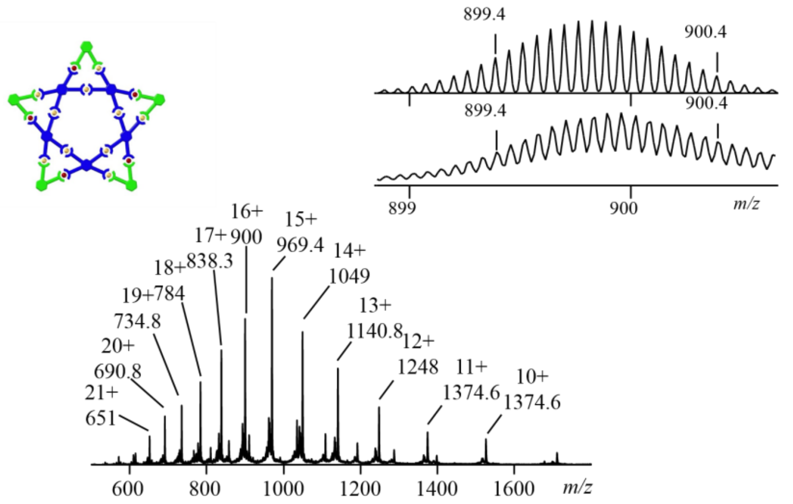

Supplementary Figure 48: the MS-Spectrum of $[Fe_{10}\boldsymbol{LA}_5]^{30+}$ with different charge states from +9 to +23 and measured (bottom) and calculated (top) isotope patterns.

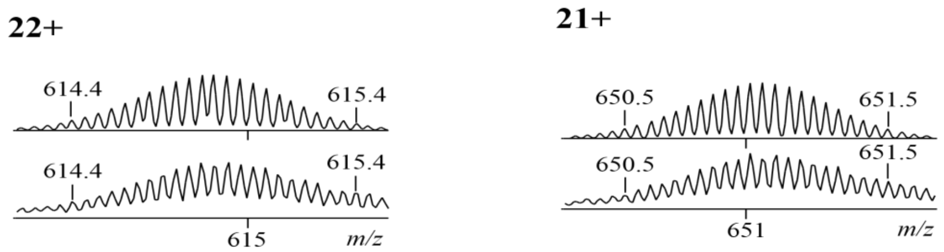

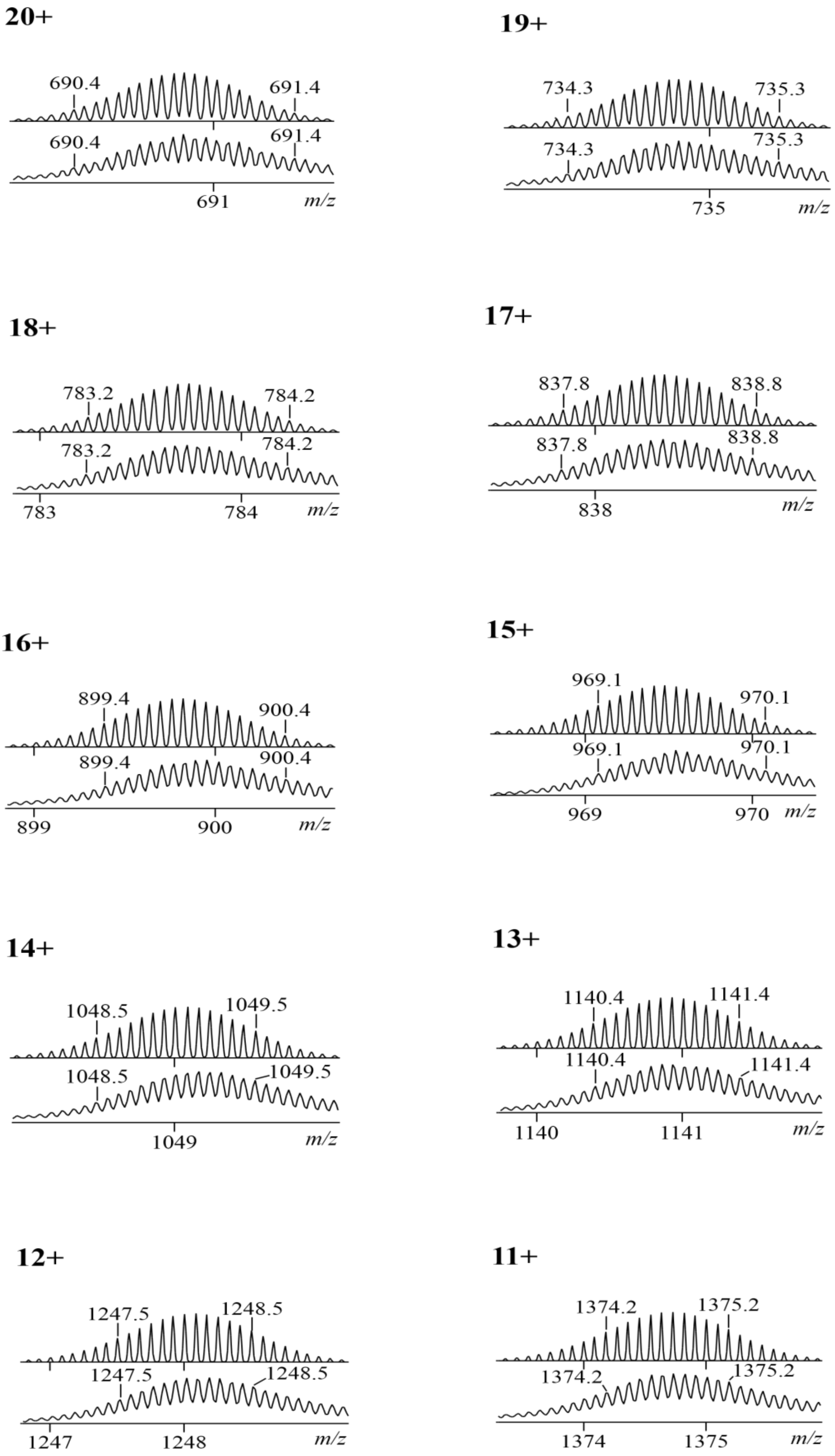

20+
690.4
691.4
690.4
691.4
691
m/z
19+
734.3
735.3
734.3
735.3
735
m/z
18+
783.2
784.2
783.2
784.2
783
784
m/z
17+
837.8
838.8
837.8
838.8
838
m/z
16+
899.4
900.4
899.4
900.4
899
900
m/z
15+
969.1
970.1
969.1
970.1
969
970
m/z
14+
1048.5
1049.5
1048.5
1049.5
1049
m/z
13+
1140.4
1141.4
1140.4
1141.4
1140
1141
m/z
12+
1247.5
1248.5
1247.5
1248.5
1247
1248
m/z
11+
1374.2
1375.2
1374.2
1375.2
1374
1375
m/z

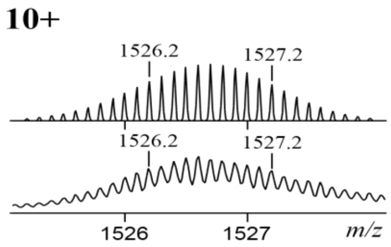

Supplementary Figure 49: Measured (bottom) and calculated (top) isotope patterns for the different charge states observed from $[\mathit{Fe}_{10}\boldsymbol{LA}_5]^{30+}$ (PF_6^- as counterion).

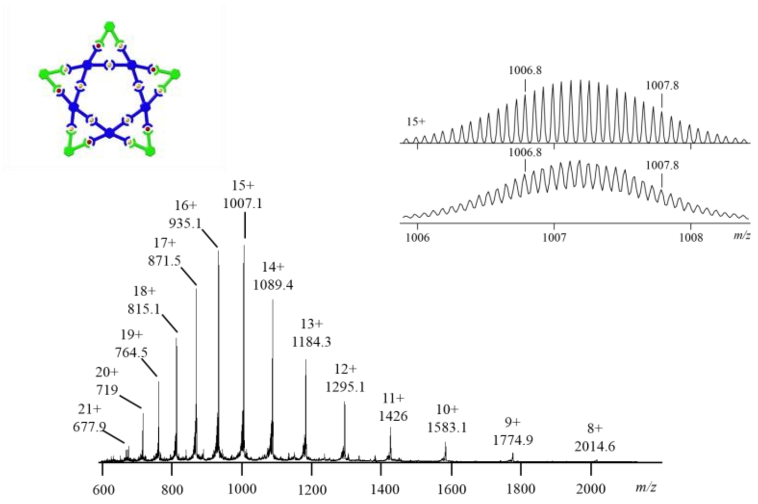

Supplementary Figure 50: the MS-spectrum of $[\mathit{Cd}_{10}\boldsymbol{LA}_5]^{30+}$ with different charge states from +8 to +21 and measured (bottom) and calculated (top) isotope patterns.

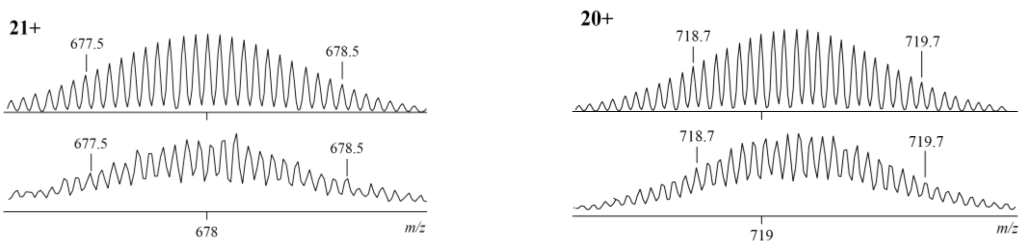

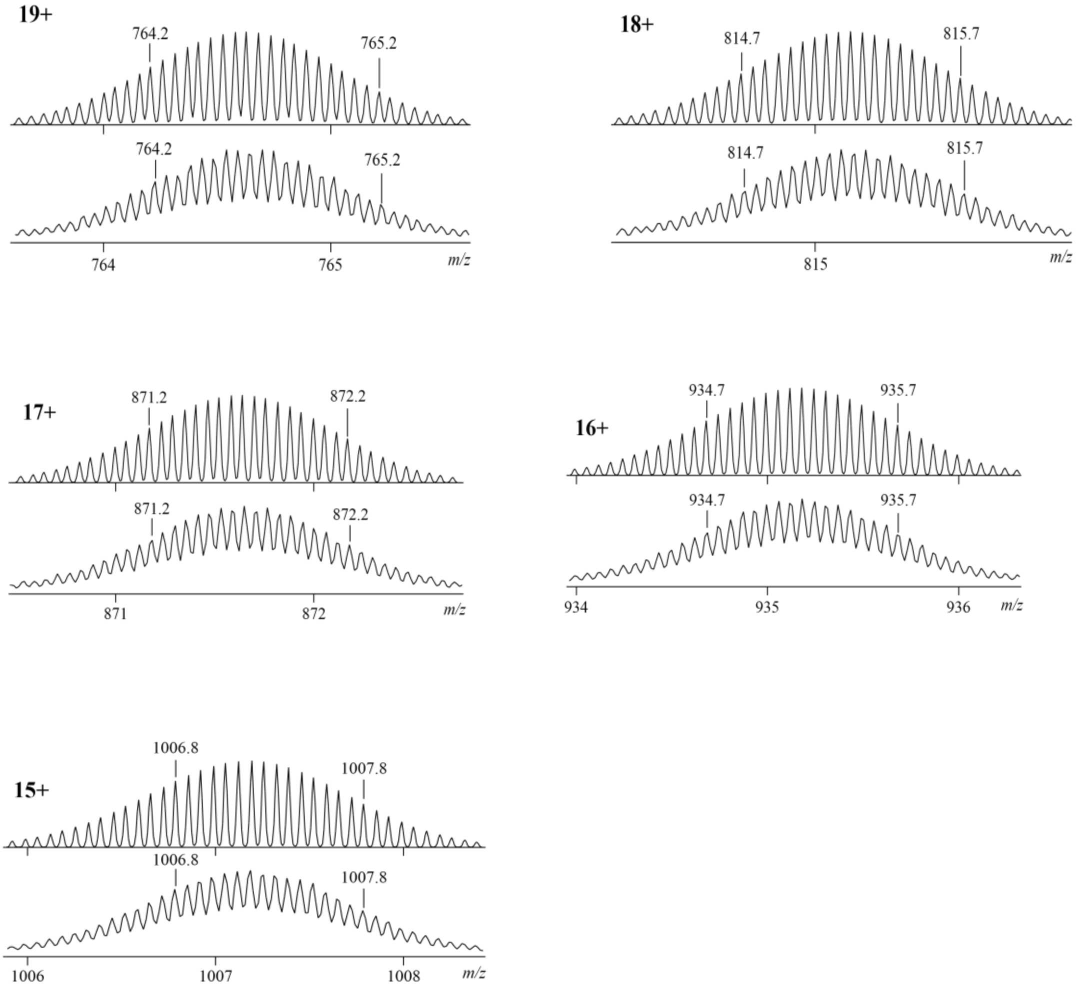

Supplementary Figure 51: the Measured (bottom) and calculated (top) isotope patterns for the different charge states observed from $[Cd_{10}\boldsymbol{LA}_5]^{30+}$ (PF_6^- as counterion).

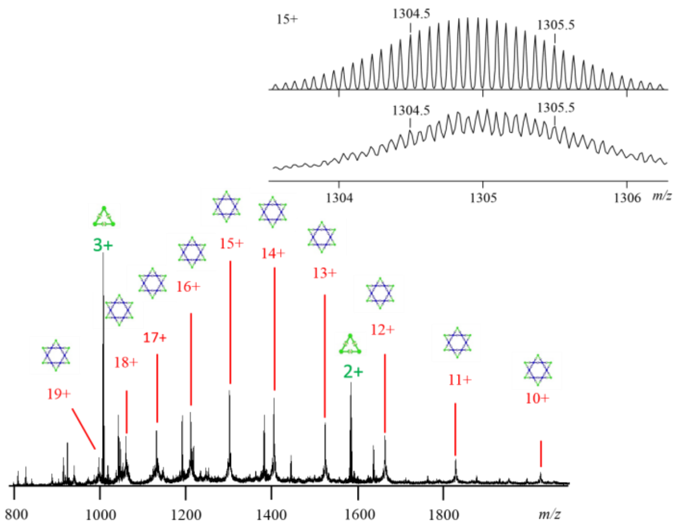

Supplementary Figure 52: the MS-Spectrum of $[Cd_{12}\boldsymbol{V}_3(\boldsymbol{LB})_3]^{36+}$ with different charge states from +10 to +19 and impurities triangle of $[Cd_3\boldsymbol{V}_3]^{6+}$. Inset: measured (bottom) and calculated (top) isotope patterns of $[Cd_{12}\boldsymbol{V}_3(\boldsymbol{LB})_3]^{36+}$.

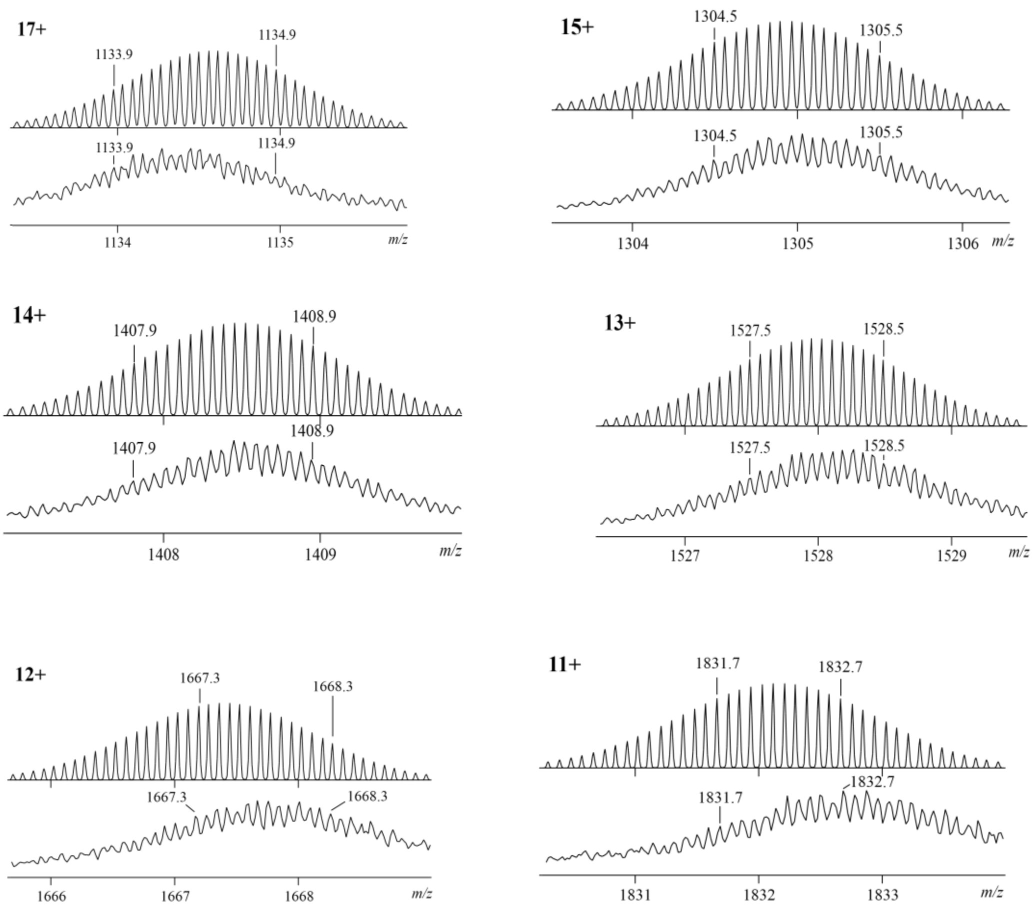

Supplementary Figure 53: the Measured (bottom) and calculated (top) isotope patterns of

$[Cd_{12}\boldsymbol{V}_3(\boldsymbol{LB})_3]^{36+}$ for the different charge states (PF_6^- as counterion, only isotopic patterns of $[Cd_{12}\boldsymbol{V}_3(\boldsymbol{LB})_3]^{36+}$ were presented; the isotopic patterns of the metallo-triangle $[Cd_3\boldsymbol{V}_3]^{6+}$ was consistent with the published results).

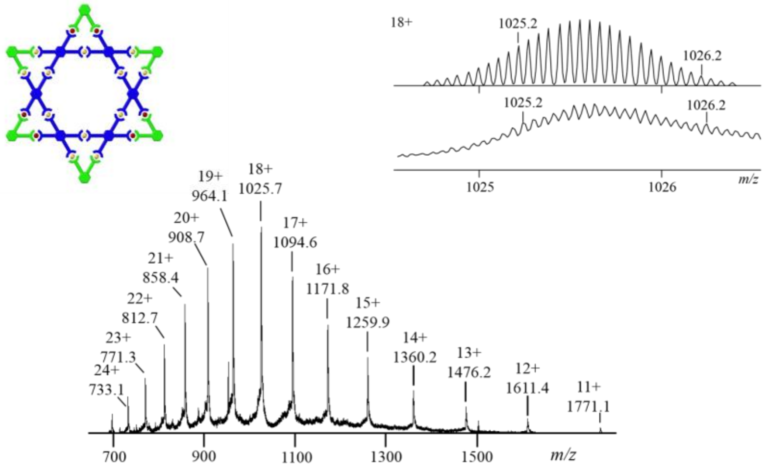

Supplementary Figure 54: the MS-spectrum of $[Fe_{12}\boldsymbol{V}_3(\boldsymbol{LB})_3]^{36+}$ with different charge states from +11 to +25 and the measured (bottom) and calculated (top) isotope patterns.

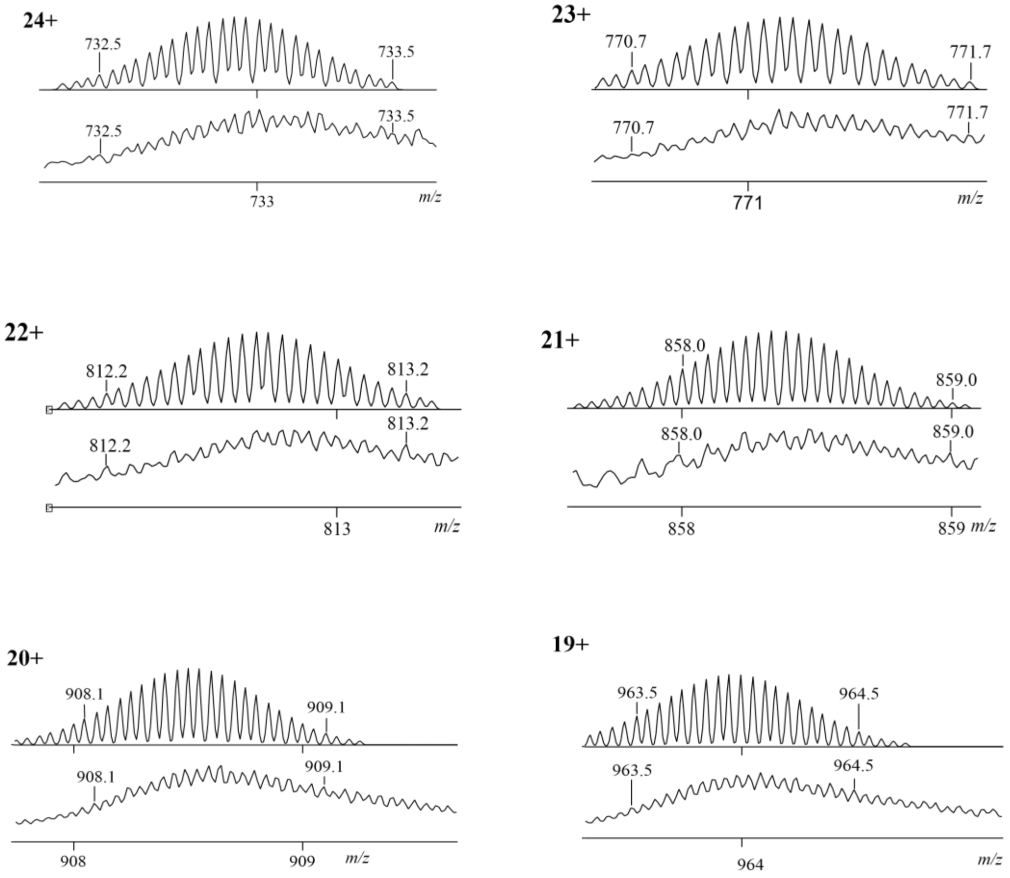

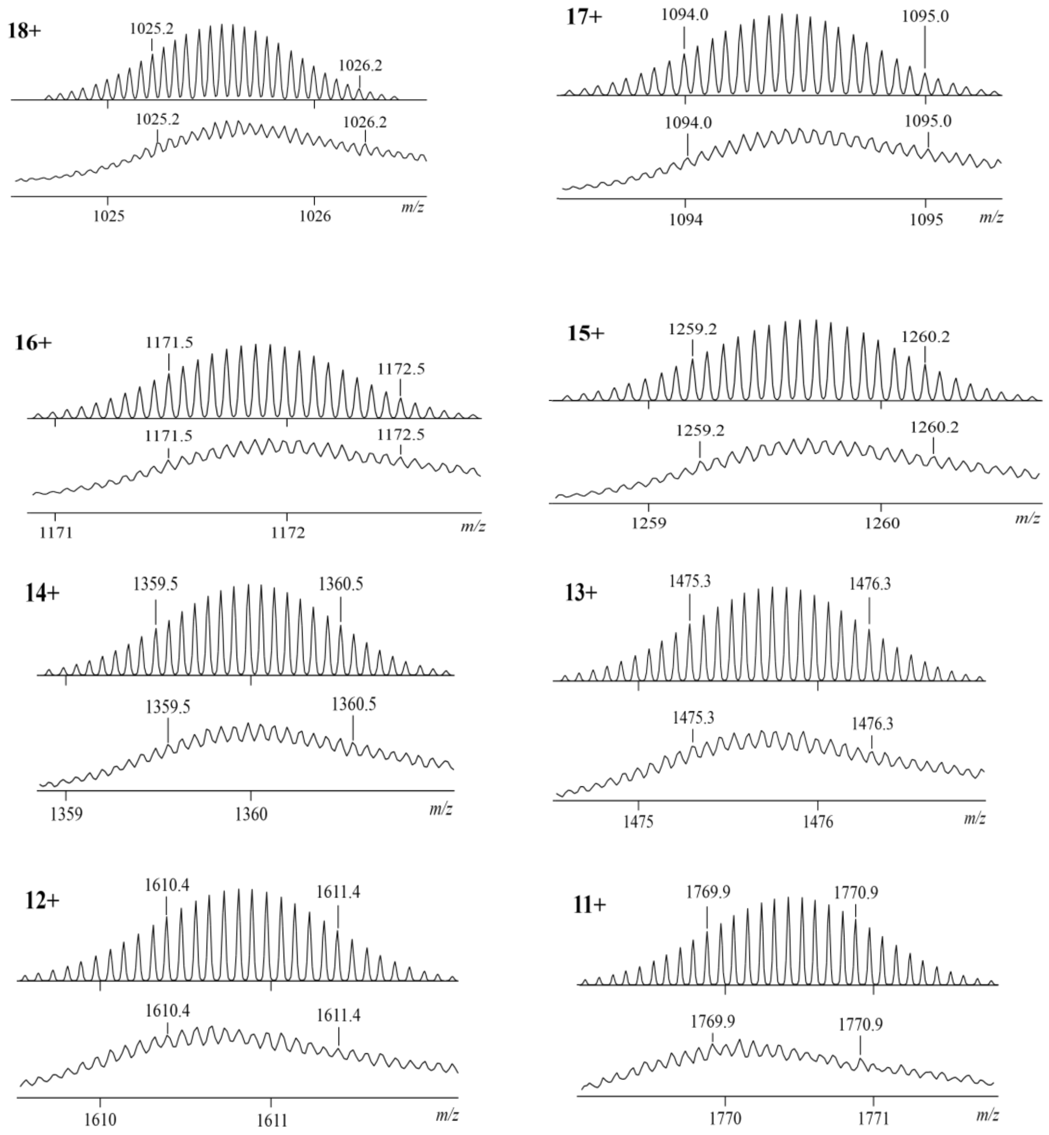

Supplementary Figure 55: the Measured (bottom) and calculated (top) isotope patterns for the different charge states observed from [$\textit{Fe}_{12}\boldsymbol{V}_3(\boldsymbol{LB})_3$]$^{36+}$ (PF_6^- as counterion).

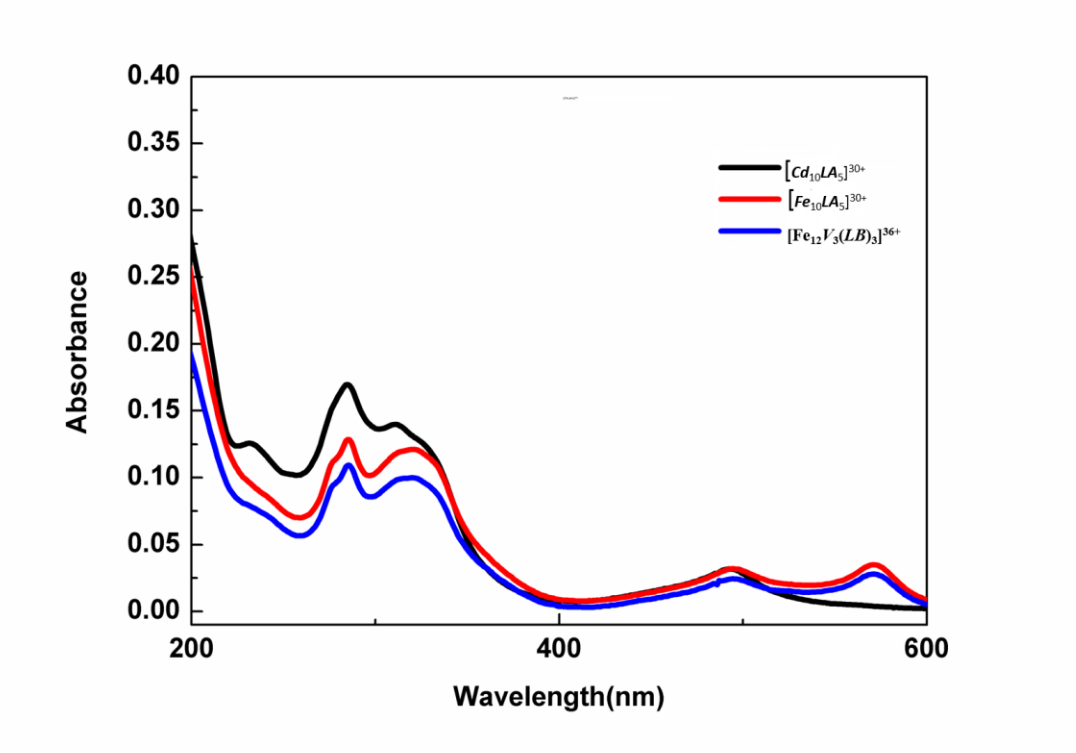

Supplementary Figure 56: The UV-vis absorption spectrum of $[\boldsymbol{Cd_{10}LA_5}]^{30+}$, $[\boldsymbol{Fe_{10}LA_5}]^{30+}$, $[\boldsymbol{Fe_{12}V_3(LB)_3}]^{36+}$ with the concentration ~10^{-6} M in CH_3CN.

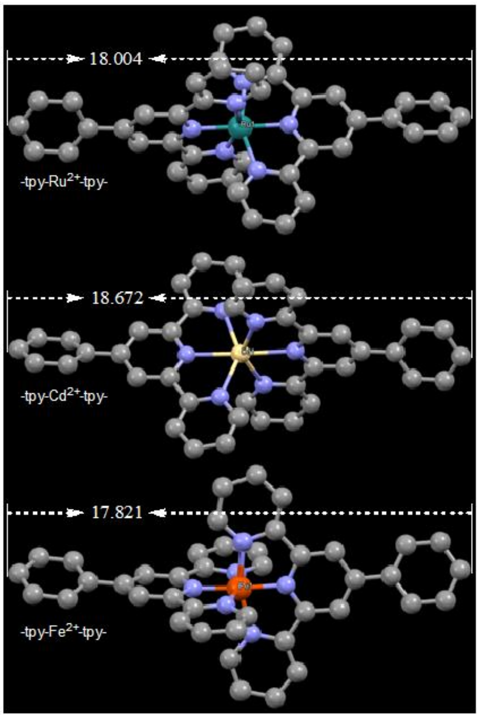

Supplementary Figure 57: Single crystal structures of tpy-Ru-tpy, tpy-Cd-tpy and tpy-Fe-tpy, and their measured lengthes.[S5]

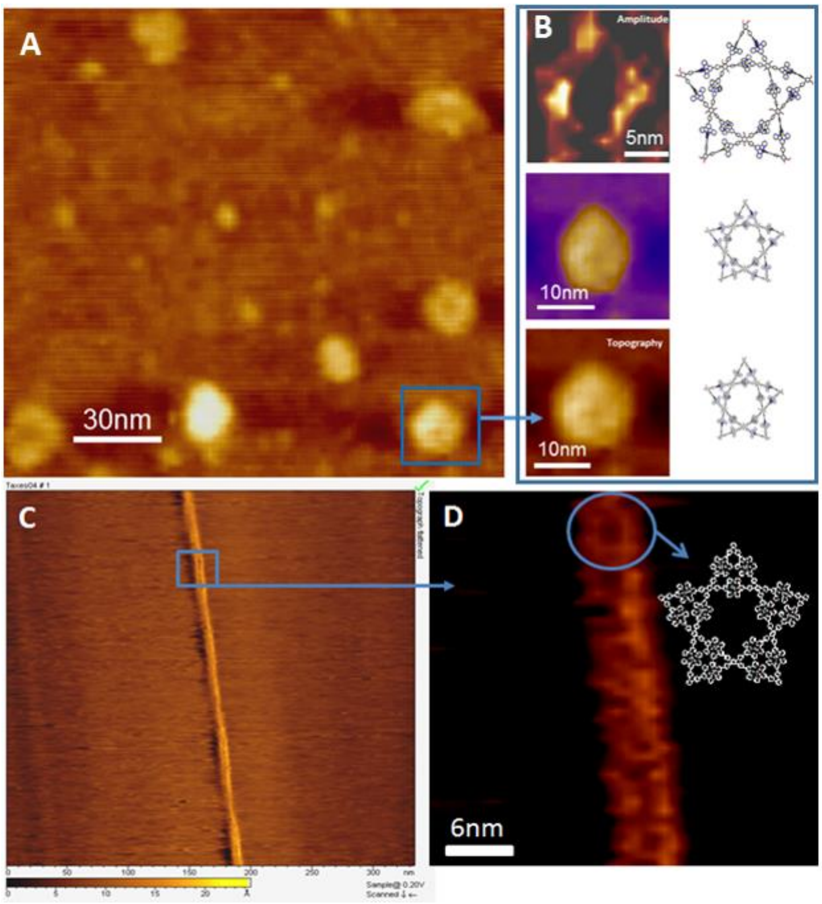

Supplementary Figure 58: A and B: AFM images of metallo-pentagram; C and D: STM images of metallo-pentagram.

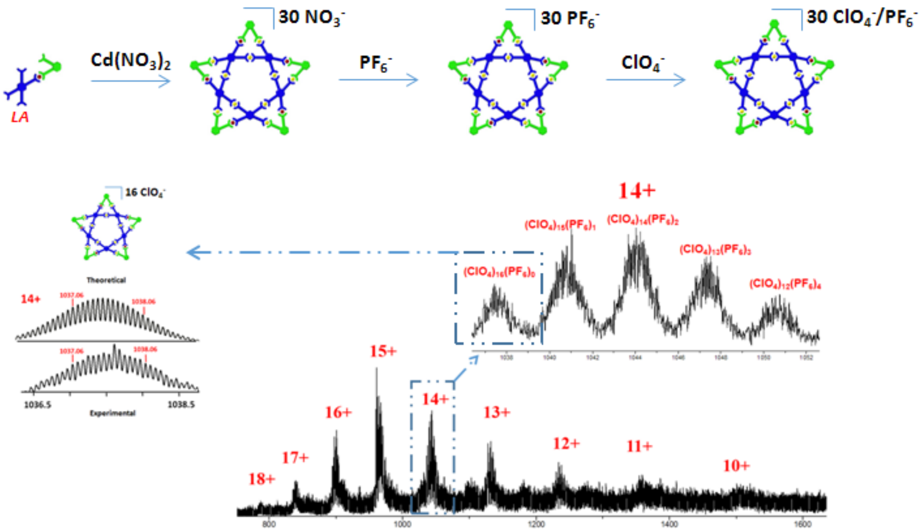

Supplementary Figure 59: High-resolution ESI-MS of pentagram $[Cd_5LA_5]^{30+}$ with different anions of NO_3^-, PF_6^- and/or ClO_4^-.

Supplementary References:

1. A. Schultz, X. Li, B. Barkakaty, C. N. Moorefield, C. Wesdemiotis and G. R. Newkome. *J. Am. Chem. Soc.* **2012**, *134*, 7672−7675

2. Y. Li, Z. Jiang, J. Yuan, D. Liu, T. Wu, C. N. Moorefield, G. R. Newkome and P. Wang. *Chem. Commun.,* **2015**, *51*, 5766-5769.

3. T. Bauer, D. Schlüter, J. Sakamoto. *Synlett*, **2010**, *06*, 877-880.

4. J. M. Ludlow III, Z. Guo, A. Schultz, R. Sarkar, C. N. Moorefield, C. Wesdemiotis, and G. R. Newkome. *Eur. J. Inorg. Chem.* **2015**, 5662–5668.

5. E. C. Constable, C. E. Housecroft, E. A. Medlycott, M. Neuburger, F. Reinders, S. Reymann and S. Schaffner, *Inorg. Chem. Commun.*, **2008**, *11*, 805 (for **tpy-Ru-tpy**); N. W. Alcock, P. R. Barker, J. M. Haider, M. J. Hannon, C. L. Painting, Z. Pikramenou, E. A. Plummer, K. Rissanen and P. Saarenketo, *J. Chem. Soc*., *Dalton Trans*., **2000**, 1447–1461 (for **tpy-Cd-tpy**); J. C. McMurtrie and I. Dance, *Cryst. Eng. Commun.*, **2009**, *11*, 1141 (for **tpy-Fe-tpy**).

6. I. Horcas, R. Fernández, J. M. Gómez-Rodríguez, J. Colchero, J. Gómez-Herrero, A. M. Baro, *Rev. Sci. Instrum.* **2007**, *78*, 013705.
